# Effects of an 18-month meditation training on dynamic functional connectivity states in older adults: Secondary analyses from the Age-Well randomized controlled trial

**DOI:** 10.1162/IMAG.a.33

**Published:** 2025-06-10

**Authors:** Sacha Haudry, Sophie Dautricourt, Julie Gonneaud, Brigitte Landeau, Vince Daniel Calhoun, Robin de Flores, Geraldine Poisnel, Salma Bougacha, Elizabeth Kuhn, Edelweiss Touron, Léa Chauveau, Francesca Felisatti, Cassandre Palix, Denis Vivien, Vincent de la Sayette, Antoine Lutz, Gaël Chételat

**Affiliations:** Normandy University, UNICAEN, INSERM, U1237, PhIND “Physiopathology and Imaging of Neurological Disorders”, Neuropresage Team, Caen, France; Memory center of Lyon, University Hospital Lyon, Lyon, France; Tri-institutional Center for Translational Research in Neuroimaging and Data Science (TReNDS), Georgia State University, Georgia Institute of Technology, Emory University, Atlanta, GA, United States; German Center for Neurodegenerative Diseases (DZNE) Bonn, Bonn, Germany; Department of Cognitive Disorders and Old Age Psychiatry, University Hospital Bonn, Bonn, Germany; Normandy University, UNICAEN, INSERM, U1237, PhIND “Physiopathology and Imaging of Neurological Disorders”, Institut Blood & Brain @ Caen, Cyceron, Caen, France; Département de Recherche Clinique, CHU Caen-Normandie, Caen, France; Service de Neurologie, Caen-Normandie University Hospital, CHU, Caen, France; Université Claude Bernard Lyon 1, CNRS, INSERM, Centre de Recherche en Neurosciences de Lyon CRNL U1028 UMR5292, EDUWELL, Bron, France

**Keywords:** meditation, dynamic functional connectivity, non-pharmacological intervention, AD risk factors, AD protective factors, neuroimaging

## Abstract

Meditation training in older adults has been proposed as a non-pharmacological intervention to promote healthy aging and lower the risks of developing Alzheimer’s disease (AD). Resting-state dynamic functional network connectivity (dFNC) highlighted two brain states, the “strongly connected” and “default mode network (DMN)-negatively connected” states, associated with protective factors for dementia including AD, and two states, the “weakly connected” and “salience-negatively connected” states, associated with risk factors for dementia. In this study, we aimed at assessing the impact of an 18-month meditation training on dFNC states in older adults. One hundred and thirty-five healthy older adults were randomized (1:1:1) to 18-month meditation training, 18-month non-native language training, or no intervention. dFNC of the DMN, salience, and executive control networks was assessed in 124 individuals using a sliding window framework, and states were obtained by k-means clustering. Linear mixed models evaluated the change in time spent in different connectivity “states” and the number of transitions between states for each group and between groups. Only participants in the meditation group transitioned significantly more between states (p = 0.008, d = 0.52), with a significant between-group difference with the non-native language training group (p = 0.001). Moreover, only the meditation group showed a change in time spent in specific states, spending less time in the “weakly connected” state (p = 0.009, d = -0.44) and more time in the “strongly connected” state (p = 0.03, d = 0.46), but there was no difference between groups. Brain states at rest were significantly impacted by an 18-month meditation intervention, with increased number of transitions between states, an increased time spent in the “strongly connected” state, and decreased time spent in the “weakly connected” state. While only the first change differed significantly between groups, these results suggest a beneficial effect of meditation through a reduction in dFNC metrics associated with AD risk factors and an increase in dFNC metrics associated with protective factors. However, the absence of a significant group-by-time interaction for time spent in states, the small effect sizes, and the fact that the sample size was not powered for this outcome limit the interpretation of the findings. Additionally, unmeasured factors such as genetic predisposition and lifestyle could have influenced the results. Future studies should identify the specific active mechanisms of meditation underlying these effects to optimize interventions. Trial Registration: The Age-Well randomized controlled trial (RCT) was approved by the local ethics committee (CPP Nord-Ouest III, Caen; trial registration number: EudraCT: 2016-002441-36; IDRCB: 2016-A01767-44; ClinicalTrials.gov Identifier: NCT02977819; registration date: 2016-11-25).

## 1 Introduction

Aging is a natural phenomenon affecting the organism at all levels—molecular, cellular, structural, behavioral and psychological (Sperduti et al., 2017). It is associated with an increased risk of developing diseases—including neurodegenerative disorders such as Alzheimer’s disease (AD) (Gaspar-Silva et al., 2023). Age-related changes notably affect brain structure and function, including the functional connectivity within and between brain networks—which has been investigated in many studies on aging (Dennis & Thompson, 2014;Ferreira & Busatto, 2013;Hrybouski et al., 2021). In recent years, interest has grown in dynamic functional network connectivity (dFNC), a technique developed as an alternative to the assumption of temporally “static” functional connectivity over the duration of the acquisition, to account for the fact that functional connections vary over time (Allen et al., 2012,^2014^). This novel approach allows us to assess the changes in functional connectivity over short periods of time through the identification and transition of specific connectivity configurations (hereby named “states”) (Allen et al., 2014;Calhoun et al., 2014). The links between dFNC and cognition are not fully understood, as findings diverge. Some studies show that dFNC relates to cognitive processes and performance, with variations in dFNC linked to fluctuations in arousal and cognitive demands, reflecting cognitive abilities (Cohen, 2018;Hutchison, Womelsdorf, Allen, et al., 2013;Vidaurre et al., 2017;Yamashita et al., 2021). This suggests that dFNC may be a valuable tool for understanding how brain function adapts to cognitive challenges and aging. However, other studies suggest that dFNC can occur independently of conscious cognitive processing, as dynamic fluctuations have been observed in subjects under anesthesia across different species (Hutchison, Womelsdorf, Gati, et al., 2013;Keilholz et al., 2013;Majeed et al., 2011).

dFNC characteristics, that is, time spent in specific states or number of transitions between states, were found to be sensitive to aging and AD. Specifically, older adults were found to spend more time in a state characterized by weak interactions between networks compared with young adults (Tian et al., 2018;Wu et al., 2023;Xia et al., 2018). Similar findings have been reported when comparing patients on the AD continuum with controls (Chen et al., 2021;Du et al., 2020), although these results have not been consistently observed in all studies (Fu et al., 2019;Gu et al., 2020). Interestingly, it has been shown that specific dFNC states in cognitively unimpaired older adults were differentially associated with either risk or protective factors for dementia. Thus, in a previous study, we found that 4 dFNC states could be identified in a population of 127 cognitively unimpaired older adults from the baseline data of the Age-Well cohort: a “weakly connected,” a “salience (SN)-negatively connected,” a “strongly connected,” and a “default mode network (DMN)-negatively connected” states (Dautricourt et al., 2022). We showed that the first two states were associated with risk factors for AD (less early- and midlife cognitive activities, higher cardiovascular risk factors, and more depressive symptoms) while the last two states were linked to protective factors (higher early- and midlife cognitive activities and lower cardiovascular risk factors) (Dautricourt et al., 2022). Given that dFNC states appear to reflect AD risk and protective factors, investigating whether interventions such as meditation can modulate these states is of particular interest.

Meditation, a form of mental training aimed at improving one’s core psychological capacities such as attention and emotional regulation (Tang et al., 2015), is thought to be protective against neurodegenerative diseases and notably AD (Chételat et al., 2018;Lutz et al., 2021). Indeed, meditation training has been found to be associated with reduced depression, stress, anxiety, and sleep disturbances—all linked to increased risk of AD, and to have beneficial effects on cognition, brain structure, and function (Chételat et al., 2018;Hatch et al., 2023;Lutz et al., 2021;Reangsing et al., 2022). Moreover, meditation training has been associated with changes in functional connectivity in the general population (Sezer et al., 2022) and in older participants (Cotier et al., 2017) and is thus thought to have the potential to impact dFNC. Changes in brain dFNC states might be one of the mediators of the beneficial effect of meditation training in aging notably in reduction of dementia risk. A previous study assessed the effect of a 6-week intervention mixing meditation, physical exercise, discussion, and readings, and did indeed find changes in dFNC parameters in young adults (Mooneyham et al., 2017), but no study has yet focused on the specific effect of a long-term meditation intervention on dFNC in older adults.

Our goal in this study was to assess the impact of an 18-month meditation training on the dFNC parameters that we previously found to be associated with protective or risk factors for AD. To this end, we used the longitudinal data from the Age-Well clinical trial (a three-arm randomized clinical trial (RCT) comprising meditation training, foreign language training, and a no intervention group), and assessed whether the meditation training had an impact on dFNC and whether this effect was different from the control groups. Note that dFNC was not pre-registered as a primary or secondary outcome measure within the clinical trial, making this analysis exploratory. Based on the Medit-Ageing model on the cognitive mechanisms likely engaged in our meditation intervention (Lutz et al., 2021), we hypothesized that participants in the meditation training group would show reduced time spent in states thought to be associated with AD risk factors and more time spent in states thought to be associated with protective factors. We anticipated that these effects would be at least partly specific to the meditation intervention, given its protective role, which is thought to stem from its dual impact on both cognitive and emotional control—unlike other cognitive training interventions, such as non-native language training.

## 2 Methods

### 2.1 General design of the study

Age-Well was a monocentric, observer-masked, RCT (Poisnel et al., 2018) with 3 parallel arms: an 18-month meditation training arm, an 18-month non-native language (English) training arm, and a no intervention arm. Participants underwent multimodal assessments including cognitive, behavioral, neuroimaging, and biological assessments. Data reported here correspond to post-intervention versus baseline (pre-intervention) resting-state functional magnetic resonance imaging (fMRI) data. While a second visit (mid-intervention) occurred at mid-intervention, no imaging acquisition was conducted during this session. The Age-Well RCT was approved by the local ethics committee (CPP Nord-Ouest III, Caen; trial registration number: EudraCT: 2016-002441-36; IDRCB: 2016-A01767-44; ClinicalTrials.gov Identifier: NCT02977819; registration date: 2016-11-25). All participants gave their written informed consent prior to the examinations. The study was conducted in accordance with the Declaration of Helsinki.

### 2.2 Participants

A total of 137 cognitively unimpaired older adults were randomized. Out of the 137 participants, 2 were excluded from the trial after randomization: 1 was diagnosed with amyotrophic lateral sclerosis and 1 had an experience of a head trauma with loss of consciousness for more than 1 h. In addition, another participant died before the end of the trial, and 10 were excluded from the fMRI analyses due to quality check failure (excessive head movements). The flow chart is represented inFigure 1and the demographic characteristics of participants included in the longitudinal analyses are summarized inTable 1. The Age-Well trial protocol has been described in a previous publication (Poisnel et al., 2018), and is detailed in theSupplementary Methods. Briefly, participants were recruited from the general population between November 2016 and March 2018, in Caen (France). They were all over 65 years old, had completed at least 7 years of education, were native French speakers, performed within the normal range for age and educational level on standardized cognitive tests, and had no major neurological or psychiatric disorder. In addition, participants had no strong preference or aversion for an intervention group, had no present or past regular or intensive practice of meditation or comparable practices, and did not speak fluent English. All participants gave their written informed consent prior to the examinations.

**Fig. 1.**
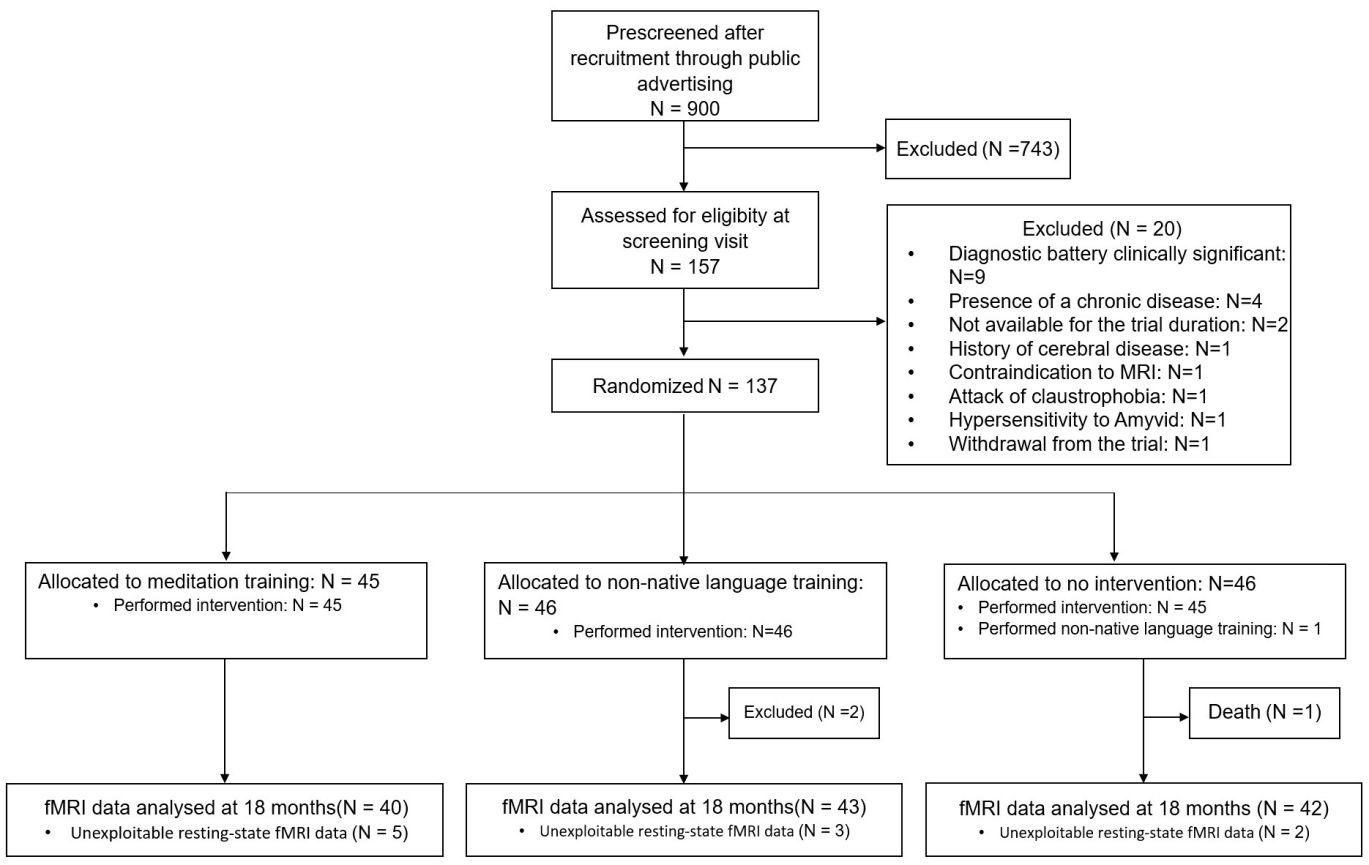
CONSORT diagram of study enrollment and randomization. Abbreviations: MRI = magnetic resonance imaging, fMRI = functional magnetic resonance imaging.

**Table 1.** Demographic characteristics at baseline of participants included in the longitudinal study.

Characteristics	Meditation training	Non-native language training	No intervention
Sample size	40	43	41
Age, years	69.5 ± 3.81	70.3 ± 4.61	68.0 ± 2.59
Education, years	13.1 ± 3.17	12.1 ± 3.02	14.3 ± 2.92
Sex, N M/F (%)	11/29 (27.5/72.5)	19/24 (44.2/55.8)	16/25 (39/61)
MMSE	28.88 ± 1.16	29.02 ± 0.99	29.22 ± 0.94

No statistical tests were conducted to compare baseline demographic characteristics between groups. Given the randomization procedure, no significant differences were expected, and no major clinical differences in demographics were observed between groups. Values are mean ± SD unless otherwise stated.

Abbreviations: MMSE = Mini Mental State Examination.

### 2.3 Randomization and blinding

Following baseline visit, participants were randomly assigned (1:1:1) to the 18-month meditation training (n = 45), 18-month non-native language training (n = 46), or no intervention arms (n = 45) according to a randomization list with permuted blocks of varying size (6 and 9). The randomization list was generated centrally by the trial statistician at the European Clinical Trials Platform & Development group (Euclid, Bordeaux, France) prior to the start of the study. Individual allocation results were concealed in sealed envelopes. All interviewers, psychometrists, and outcome assessors involved in assessments were blind to treatment allocation. Only intervention facilitators, trial-independent statisticians, and data monitoring infrastructure staff were unmasked or partially unmasked.

### 2.4 Interventions

The meditation and non-native language training interventions were structurally equivalent in overall course length, class time, and home activities, and matched for administration, dosage, duration, and level of expertise and number of facilitators per class. For both the meditation training and the non-native language training groups, the interventions lasted 18 months and comprised 2-h weekly group sessions (ranging from 67 to 71 sessions across recruitment waves) and 1 day of intensive practice.

#### 2.4.1 Meditation training

The meditation training consisted of a structured 18-month secular program specifically developed for the Silver Santé Study, designed to enhance psychological resources for healthy aging by fostering mindfulness, kindness, and compassion (Lutz et al., 2021). The program was delivered by expert meditation instructors in Caen and was divided into two consecutive 9-month phases, the first dedicated to mindfulness meditation (MM) and the second to loving-kindness and compassion meditation (LKCM).

During the mindfulness phase, participants were guided in cultivating an attentive, non-judgmental awareness of their thoughts, emotions, and sensory experiences. The training emphasized present-moment attention and the ability to observe both positive and negative mental states without reactivity or identification. This portion of the program was directly adapted from a validated mindfulness-based intervention tailored for older adults (Zellner Keller et al., 2014).

The subsequent LKCM phase aimed to foster a positive relationship with oneself and others by addressing emotions such as self-criticism or anger and developing gratitude, care, and compassion. Participants learned to extend kindness and acceptance toward themselves, as well as progressively toward loved ones, neutral individuals, and even difficult persons, ultimately recognizing the shared human need for well-being and connection.

The integration of MM and LKCM within a single intervention is consistent with traditional meditation training models and contemporary LKCM-based interventions, which typically introduce MM first to establish attentional stability before progressing to prosocial and emotionally engaged practices (Singer & Klimecki, 2014). Emerging clinical evidence suggests that LKCM may complement MM by further enhancing emotional balance, purpose in life, and social support—factors particularly relevant for aging populations (Donald et al., 2019;Fredrickson et al., 2008;Ryff et al., 2016). Neuroimaging studies also indicate that training in MM and LKCM engages distinct but complementary neural mechanisms, supporting the rationale for their combined use (Klimecki et al., 2013;Valk et al., 2017).

Each session combined guided meditation—both seated and walking—with moments of discussion and theoretical teaching. A new educational theme was introduced each month, which was explored and practiced in subsequent sessions. Additionally, an intensive meditation day was included, allowing participants to deepen their practice through approximately 5 h of meditation.

Full details of the intervention, including session structure and theoretical foundations, are available in theSupplementary Materialsand in the Age-Well study protocol paper (Poisnel et al., 2018).

#### 2.4.2 Non-native language training

The non-native language training program is a cognitively stimulating program. It consists of English exercises designed to strengthen each participant’s abilities to understand, write, and speak English. Sessions were tailored to baseline English proficiency levels and were held by mixing oral comprehension and expression activities to emphasize the acquisition of new vocabulary and grammatical structures. Non-native language training was chosen based on evidence suggesting its potential cognitive benefits in aging, which may help delay cognitive decline (Antoniou & Wright, 2017;Antoniou et al., 2013;Ware et al., 2021). Full details are given in theSupplementary Materialsand in the protocol paper of the Age-Well study (Poisnel et al., 2018).

#### 2.4.3 No intervention group

Participants in the no intervention group were requested not to change their habits and to continue living as they did before engaging in the study and until the end of it. They were specifically asked not to engage in meditation or non-native language training during the 18-month period of the study (Poisnel et al., 2018).

### 2.5 Neuroimaging acquisition

#### 2.5.1 Resting-state fMRI

fMRI volumes were collected during resting state in the three groups (at baseline [pre-intervention] and post-intervention in the meditation training, non-native language training, and no intervention groups). fMRI volumes were obtained using an interleaved 2D T2* SENSE echo planar imaging (EPI) sequence designed to reduce geometric distortions using parallel imaging, short echo time, and anisotropic voxels (2D-T2*-FFE-EPI axial, SENSE = 2.5; repetition time = 2400 ms; echo time = 30 ms; flip angle = 85°; 44 slices with no gap; slice thickness = 2.8 mm; field of view = 200 x 200 mm^2^; in-plane resolution = 2.5 x 2.5 mm^2^; 200 volumes, acquisition time = 8’). Subjects were equipped with earplugs and their heads were stabilized with foam pads to minimize head motion. During this acquisition, subjects were asked to keep their eyes closed, to not fall asleep, and to let their thoughts come and go freely. Sample fMR images before processing are available in the Supplementary Materials (Supplementary Fig. S1).

#### 2.5.2 Anatomical magnetic resonance imaging

All participants also underwent a structural magnetic resonance imaging (MRI). A high-resolution T1-weighted anatomical volume was first acquired using a 3D fast field echo sequence (3D-T1-FFE sagittal; repetition time = 7.1 ms; echo time = 3.3 ms; flip angle = 6°; 180 slices with no gap; slice thickness = 1 mm; field of view = 256 x 256 mm^2^; in plane resolution = 1 x 1 mm^2^). A high-resolution T2-weighted Fluid Attenuated Inversion Recovery (FLAIR) anatomical volume was also collected (3D-IR sagittal; TR/TE/TI = 4800/272/1650 ms; flip angle = 40°; 180 slices with no gap; slice thickness = 1 mm; field of view = 250 x 250 mm^2^; in-plane resolution = 0.98 x 0.98 mm^2^). MRI-T1 data were segmented using FLAIR images to improve the delineation between gray matter and cerebrospinal fluid, then normalized to the Montreal Neurological Institute (MNI) space using both the T1-weighted and FLAIR images, along with the segment routine implemented in the Statistical Parametric Mapping software (SPM12) (Ashburner & Friston, 2005). A gray matter mask at the group level was obtained to be later used in the spatially constrained independent component analysis (ICA) using previously published methods (La Joie et al., 2014).

### 2.6 Preprocessing of resting-state fMRI

Sections 2.6 to 2.9 describing all steps of the dFNC methodology from preprocessing to the cluster analysis are similar to that described in the paper byDautricourt et al. (2022). Individual resting-state fMRI datasets were first checked for motion artifacts using the TSDiffAna routine (https://github.com/silverlab/megavista/blob/master/users/Rachel/tsdiffana/tsdiffana.m). Briefly, a variance volume was created for each subject to check that most signal variability was restricted to the cortex. As dynamic functional analyses can be impacted by head motion, datasets showing evidence for significant movements (>3 mm translation or 1.5° rotation) associated with image artifacts and/or an abnormal variance distribution were excluded from subsequent analyses (see also flow chart –Fig. 1). The data were then processed using SPM12. First, slice timing correction was applied to account for timing differences in slice acquisition. This was followed by realignment of the images to the first volume to correct for head motion during scanning. Noise was identified in the connectivity maps, so the images were decomposed into 50 components using ICA with FMRIB Software Library (FSL)-s Melodic tool. Noisy components were removed based on visual inspection. Resting-state fMR images were denoised by removing the visually identified noisy components from an ICA with melodic allowing to decompose the signal in 50 components. The denoised EPI volumes were then co-registered on the corresponding T1-weighted MR images, spatially normalized within the native space to correct for distortion effects, normalized to the MNI space, applying the normalization parameters of the anatomical MRI, and smoothed with a 4 mm full-width at half-maximum Gaussian kernel.

### 2.7 Identification of intrinsic connectivity networks

After data preprocessing, resting-state fMRI data of all participants were analyzed using fully automated spatially constrained ICA pipeline (Du & Fan, 2013) implemented in the Group ICA Of fMRI Toolbox (GIFT;https://github.com/trendscenter/gift) (Calhoun et al., 2001). Spatially constrained ICA was performed using an ICA template (Shirer et al., 2012), available to download athttps://greiciuslab.stanford.edu/resources. This template represents large-scale networks and is divided into 14 components which were utilized to decompose the data into these specific components. Among the 14 components, we selected 7 ICA components recapitulating the 3 intrinsic functional networks most involved in mindfulness meditation, that is, the DMN, the SN, and the executive control network (ECN) (Hasenkamp et al., 2012), which are represented inSupplementary Figure S2. Anatomical description of each component is detailed inSupplementary Table S1. These networks were selected based on their relevance to both meditation and Alzheimer’s disease, as they are central to cognitive processes that may be modulated by mindfulness training. Moreover, this selection builds upon the work ofDautricourt et al. (2022), who also focused on these components in the baseline data of this trial, providing a basis for continuity in our study (Dautricourt et al., 2022). The advantage of using a spatially constrained ICA approach is to enhance robustness to artifacts and noise compared with single-subject ICA denoising and regression-based back reconstruction (Salman et al., 2019) and also facilitated automated component labeling and sorting. Images were masked with the gray matter mask. Following ICA, each time course was normalized using temporal normalization. Time courses were detrended, despiked using 3Ddespike, and filtered by a fifth-order Butterworth low-pass filter with a high-frequency cutoff of 0.15 Hz (Rachakonda et al., 2010).

### 2.8 Dynamic functional network connectivity

dFNC was estimated using the sliding window approach implemented in the GIFT toolbox (Iraji et al., 2020;Rachakonda et al., 2010), as described in previous publications (Allen et al., 2014;Calhoun et al., 2014). Resting-state data were divided into 182 sliding windows of 18 repetition times (43 s) size, in steps of one repetition time (2.4 s). These time windows were convolved with a Gaussian kernel with a full width at half maximum (FWHM) of 4.8 s (α = 2 repetition times), given that a window length between 30 and 60 s is suitable to estimate dFNC (Preti et al., 2017). To account for potential confounding effects, we regressed out six head motion parameters from realignment to remove potential sources of spurious variance. Within each of these windows, we imposed an L1 norm of the precision matrix to promote sparsity, retaining only the most significant connections while eliminating weaker ones. Finally, functional connectivity matrices were transformed into z-scores using Fisher’s Z-transformation to stabilize variance prior to further analyses.

### 2.9 Clustering analysis

A k-means clustering (Lloyd, 1982) was applied to the dynamic functional connectivity matrices, comprising temporal connectivity patterns between 21 IC pairs across 182 sliding windows for each subject, to identify recurring functional connectivity patterns across time and subject space (Allen et al., 2014;Calhoun et al., 2014). Briefly, the optimal number of clusters (referred to as “states”) was determined as equal to four (k = 4), using the elbow criterion, accounting for 92.3% of the total variance (seeSupplementary Methods). Sensitivity to cluster number was assessed to check whether the results would be dependent upon this criterion. Results are presented in theSupplementary Materials. Each window of each subject was then categorized to one of these connectivity states based on the similarity with the cluster centroid using Euclidean distance to measure the similarity.

Temporal dFNC parameters were then extracted for each subject, including (1) the total time for each state (i.e. the total fraction of time the subject spent in each state) and (2) the number of transitions (i.e. the number of times the subjects switched between states).

### 2.10 Sample size

Age-Well was powered to detect an effect size of 0.75 for the trial’s coprimary outcomes (i.e., volume and perfusion of the anterior cingulate cortex and insula), with 80% power and a 2-sided type I error of 1.25% (Chételat et al., 2022). This resulted in a minimum of 126 participants (42 per arm), which was exceeded (137 total participants). Following guidance (Zhang et al., 2019), post hoc power analyses were not performed for this secondary outcome study.

### 2.11 Statistical analyses

Since participants were randomized to trial arms, no statistical tests (e.g., t-tests) were conducted to compare baseline demographic characteristics such as age and gender between groups, in line with the statistical analysis guidelines for the secondary outcomes from the Medit-Ageing Consortium. However, it is important to note that there were no major clinical differences in demographic or clinical characteristics between the arms (Chételat et al., 2022). The different dFNC states were first described and the links between the dFNC parameters assessed with Pearson’s correlations using the*stats*package. Then, intervention effects on the dFNC parameters were evaluated by assessing within-group changes and by testing for between-group differences, that is, interactions between visit (i.e. baseline [pre-intervention] and post-intervention) and trial group (i.e. meditation, non-native language, and no intervention groups). Mixed effects models with participant-level random intercepts, estimated via restricted maximum likelihood were fitted for each measure. Because the Age-Well trial was not powered to detect interaction effects, important results may be missed if only the interaction is considered. Therefore, post hoc within-group changes and pairwise comparisons were also examined. For all analyses, baseline age, sex, and education level were included as fixed effects, with continuous variables mean-centered. Statistical analyses were performed in RStudio (version 4.0.3), using the*lme4*package for mixed effects models and*emmeans*package for within-group changes and pairwise comparisons (using the Tukey method). The interaction between group and time was also assessed using non-parametric analyses to account for the non-linear distribution of dFNC parameters. Full details about the methods and results are given in theSupplementary Materials.

To account for multiple comparisons, we applied false discovery rate (FDR) correction to the p-values obtained from testing the interactions on the dFNC parameters. Because all participants do not pass through all states, we replicated all analyses including only the participants who experienced the states (i.e. time spent in the state > 0) to ensure that the results remained consistent.

Finally, while our primary goal in this study was to assess the impact of meditation on brain states known to be associated with risk of protective factors for dementia, we performed exploratory analyses to test the alternative hypothesis that the brain state changes might be associated with changes in specific risk factors, cognitive and psycho-affective outcomes. Thus, when within-group changes in dFNC were found to be significant in the meditation training group, links between these dFNC changes and changes in risk and protective factors of dementia as well as cognitive, psycho-affective, meditation composite scores, and practice duration measures were assessed. Results are given in theSupplementary Materials.

## 3 Results

### 3.1 Dynamic connectivity states

Replicating our previous analyses of the baseline data of the Age-Well Cohort (Dautricourt et al., 2022), we identified four states from the dFNC k-means clustering when we combined baseline and post-intervention data. The connectivity matrix of each state is represented inFigure 2, while the characteristics of each state are presented inSupplementary Table S2and a few sample subjects are presented in the Supplementary Materials. The “weakly connected” state had a frequency (relative time spent in this state) of 45%, lasted on average for 22 windows (93.6 s), and was characterized by low or neutral connectivity between and within the 3 networks. The “SN-negatively connected” state had a frequency of 22%, lasted on average for 12 windows (69.6 s), and was characterized by high negative connectivity between the SN and the other networks (DMN and ECN), and a strong positive connectivity between the DMN and ECN and within each network. The “strongly connected” state had a frequency of 16%, lasted on average for 9 windows (62.4 s), and was characterized by a high positive connectivity between and within the DMN, the SN, and the ECN. The “DMN-negatively connected” state had a frequency of 17%, lasted on average for 9 windows (62.4 s), and was characterized by a high negative connectivity between the DMN and the other networks (SN and ECN) together with a strong positive connectivity between and within the SN and ECN and a weak connectivity within the DMN. We found that the total time spent in the “weakly connected” state was negatively correlated with the total time spent in all other states (SN-negatively connected: p < 0.001, r = -0.32; strongly connected: p < 0.001, r = -0.45; DMN-negatively connected: p < 0.001, r = -0.51). Moreover, the total time spent in the “SN-negatively connected” state was negatively correlated with the time spent in the “strongly connected” (p < 0.001, r = -0.24) and “DMN-negatively connected” states (p < 0.001, r = -0.44). The average number of transitions between states across subjects was 10 ± 4. The number of transitions was negatively correlated with the time spent in the “weakly connected” state (p < 0.001, r = -0.44) and positively correlated with the time spent in the “strongly connected” (p < 0.001, r = 0.41) and “DMN-negatively connected” states (p < 0.001, r = 0.28).

**Fig. 2.**
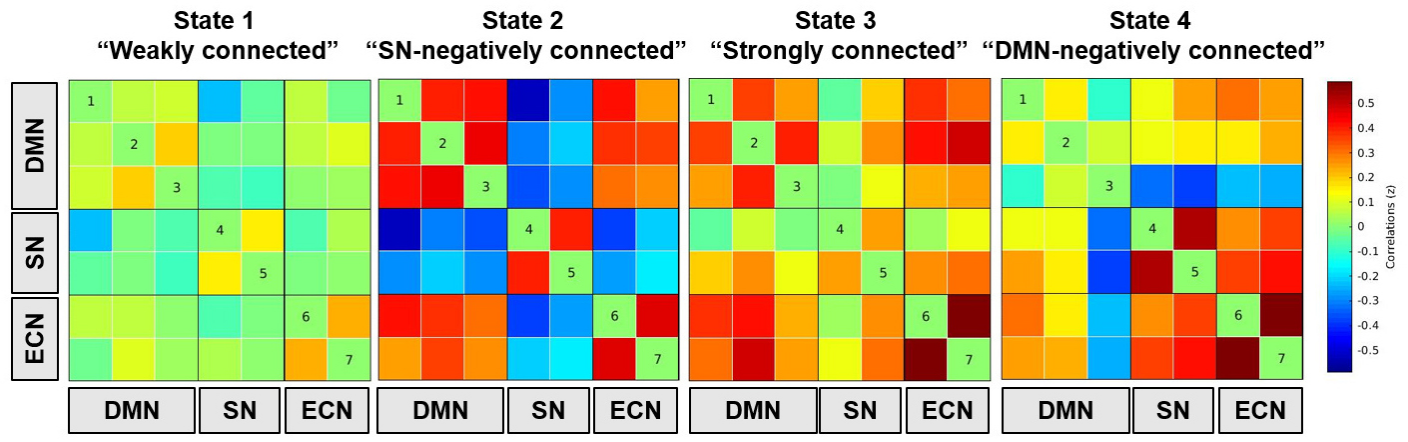
Dynamic connectivity states from the Stanford ICA templates. The four states identified from the dFNC analysis are displayed here. The color scale indicates positive (red), neutral (green), and negative (blue) connectivities between the ICA components of the DMN, SN, and ECN. States from the Stanford ICA template. Numbers 1 to 7 refer to the ICA components represented inSupplementary Figure S2. Abbreviations: DMN = default mode network, SN = salience network, and ECN = executive control network.

### 3.2 Effect of the intervention

#### 3.2.1 Total time spent in each state

Statistics of the within-group changes and linear mixed models are reported inTable 2and illustrated inFigure 3. The meditation training group showed a decrease, from baseline to post-intervention, in the total time spent in the “weakly connected” state (Cohen’s d [95% confidence interval], -0.44 [-0.8, -0.07]) and an increase in the total time spent in the “strongly connected” state (0.46 [0.01, 0.91]), while no change was found for time spent in the “SN-negatively connected” state (0.06 [-0.31, 0.44]) and “DMN-negatively connected” state (0.16 [-0.21, 0.54]). Neither the non-native language training nor the no intervention group showed any change in the total time spent in any state (Table 2). There was no group x visit interaction, that is, the change over time in the total time spent in the states was not different between the groups (Table 3). When only including the participants who experienced each specific state, the results remained similar (Supplementary Tables S3-S4andSupplementary Fig. S3), but there was a trend for a group x visit interaction for the “strongly connected” state (p = 0.07). Moreover, when using a different number of clusters or when using non-parametric analyses, results also remained similar (Supplementary Tables S5-S10).

**Table 2.** Statistics of the within-group changes of the mixed linear models.

		Standardized estimated change					
Outcome	Visit	Meditation training	p	Non-native language training	p	No intervention	p
State 1	V3-V1	-0.44 (-0.8 to -0.07)	0.009	-0.02 (-0.27 to 0.22)	0.89	-0.15 (-0.55 to 0.25)	0.4
State 2	V3-V1	0.06 (-0.31 to 0.44)	0.7	-0.04 (-0.34 to 0.26)	0.78	0.02 (-0.28 to 0.32)	0.891
State 3	V3-V1	0.46 (0.01 to 0.91)	0.03	-0.14 (-0.45 to 0.17)	0.37	0.15 (-0.19 to 0.49)	0.39
State 4	V3-V1	0.16 (-0.21 to 0.54)	0.36	0.2 (-0.13 to 0.54)	0.29	0.03 (-0.32 to 0.38)	0.86
Number of transitions	V3-V1	0.52 (0.12 to 0.93)	0.008	-0.37 (-0.68 to 0.06)	0.06	0.18 (-0.23 to 0.59)	0.3

Values are expressed as Cohen’s d (95% confidence interval). All analyses were adjusted for age, sex, and education. For within-group standardized estimated changes, positive values reflect improvement within a trial group from baseline (pre-intervention) to post-intervention; negative coefficients indicate the opposite. All p-values are adjusted for multiple comparisons with Tukey correction.

Abbreviations: state 1 = “weakly connected” state, state 2 = “SN-negatively connected” state, state 3 = “strongly connected” state, state 4 = “DMN-negatively connected” state, V1 = baseline (pre-intervention) visit, V3 = post-intervention visit.

**Table 3.** Statistics of the between-group changes of the mixed linear models.

		Interaction group x visit			Difference in change: meditation vs. non-native language training		Difference in change: meditation training vs. no intervention		Difference in change: non-native language training vs. no intervention	
Outcome	Visit	F	p	P _FDR_	Mean (95% CI)	p	Mean (95% CI)	p	Mean (95% CI)	p
State 1	V3-V1	1.74	0.18	0.36	-0.43 (-0.87. to 0.01)	0.07	0.26 (-0.71 to 0.18)	0.2	0.11 (-0.32 to 0.55)	0.62
State 2	V3-V1	0.11	0.89	0.89	0.1 (-0.34 to 0.53)	0.64	0.04 (-0.4 to 0.49)	0.85	-0.06 (-0.5 to 0.37)	0.78
State 3	V3-V1	2.5	0.086	0.34	0.48 (0.03 to 0.92)	0.03	0.22 (-0.23 to 0.66)	0.33	-0.28 (-0.72 to 0.16)	0.21
State 4	V3-V1	0.22	0.8	0.89	-0.02 (-0.46 to 0.42)	0.94	0.11 (-0.33 to 0.55)	0.6	0.14 (-0.3 to 0.57)	0.54
Number of transitions	V3-V1	5.74	0.004	0.004	0.81 (0.36 to 1.27)	0.001	0.25 (-0.2 to 0.69)	0.23	-0.46 (-0.9 to -0.02)	0.03

Values are expressed as Cohen’s d (95% confidence interval). All analyses were adjusted for age, sex, and education. For between-group difference, positive values reflect a relatively greater improvement in a trial group (from baseline [pre-intervention] to post-intervention) compared with the specified reference trial group; negative coefficients indicate the opposite. Between-group differences p-values are adjusted for multiple comparisons with Tukey correction.

Abbreviations: state 1 = “weakly connected” state, state 2 = “SN-negatively connected” state, state 3 = “strongly connected” state, state 4 = “DMN-negatively connected” state, V1 = baseline (pre-intervention) visit, V3 = post-intervention visit.

**Fig. 3.**
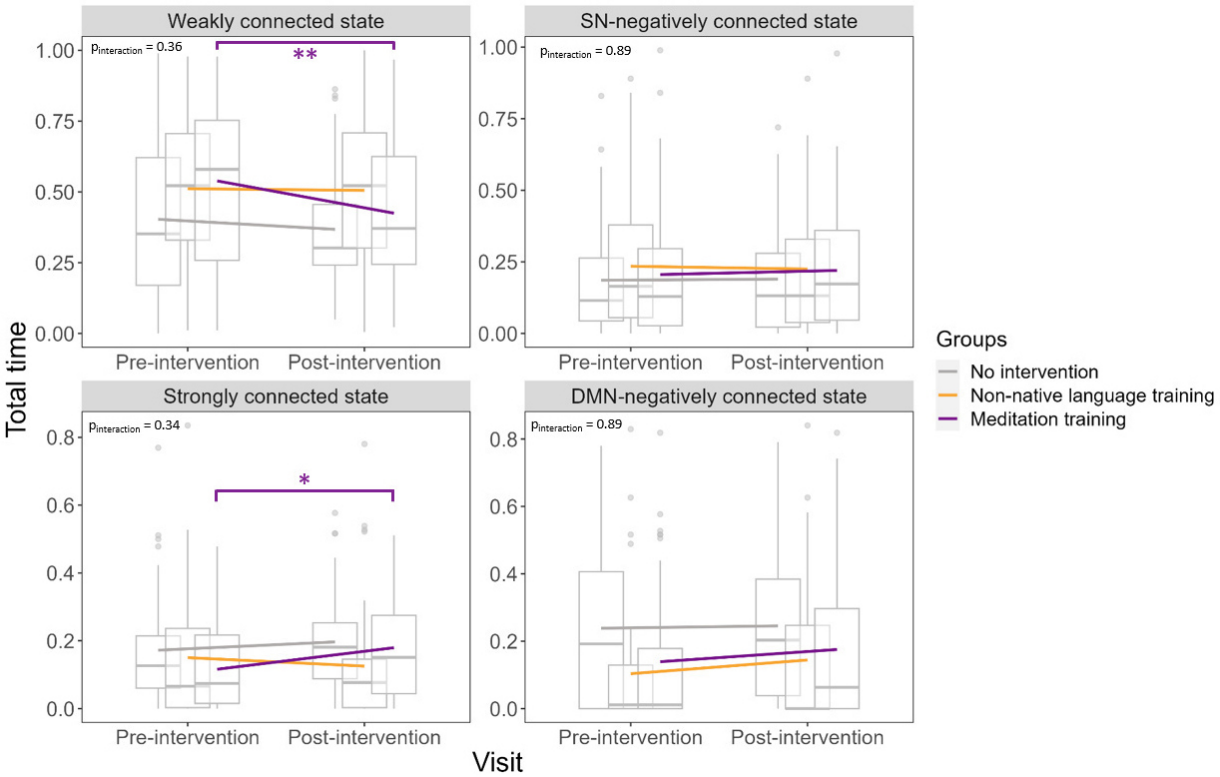
Total time spent in each state pre-/post-intervention in the three groups. Linear mixed models show no interaction between groups and visits for the total time spent in each state, controlling for age, sex, and education. Boxplots demonstrate medians and quartiles, while lines connect means. The time spent in the “weakly connected” state significantly decreased post-intervention compared with baseline (pre-intervention) in the meditation training group only. The time spent in the “strongly connected” state significantly increased post-intervention compared with baseline (pre-intervention) in the meditation training group only. The total time in each state is expressed by number of repetition time (TR) (1 TR = 2.4 s). *p < 0.05, **p < 0.01.

#### 3.2.2 Number of transitions between states

The meditation training group showed an increase, from baseline to post-intervention, in the number of transitions between states (Cohen’s d [confidence interval], 0.52 [0.12, 0.93]). Neither the non-native language training (-0.37 [-0.68, 0.06]) nor the no intervention group (0.18 [-0.23, 0.59]) showed any change in the number of transitions between states (Table 2andFig. 4). The group x visit interaction was significant (p = 0.004), and post hoc analyses showed that the number of transitions increased more in the meditation than in the non-native language training group, as well as in the no intervention group compared with the non-native language training group (Table 3andFig. 4). These results remained consistent when only including the participants who experienced each specific state, with the exception that the no intervention group was no longer superior to the non-native language training group in terms of changes in the number of transitions (Supplementary Tables S3-S4andSupplementary Fig. S4). Moreover, these results also remained similar when using a different number of clusters or when using non-parametric analyses (Supplementary Tables S5-S10).

**Fig. 4.**
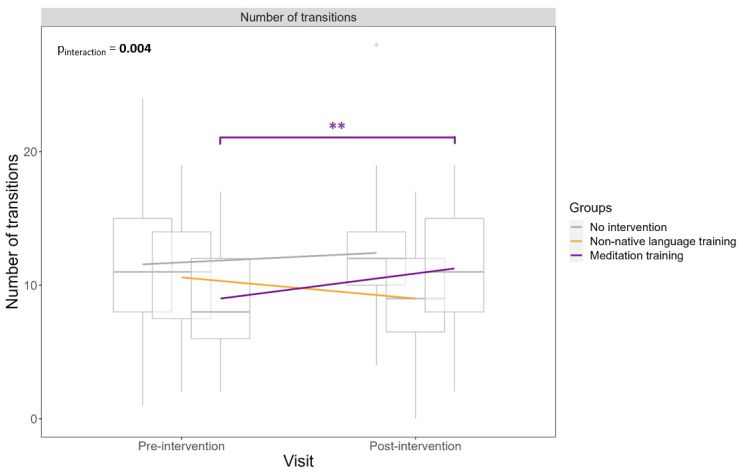
Number of transitions between states pre-/post-intervention in the three groups. Linear mixed models show a significant interaction between group and visits for the number of transitions, controlling for age, sex, and education. Boxplots demonstrate medians and quartiles, while lines connect means. The number of transitions is significantly increased post-intervention compared with baseline (pre-intervention) in the meditation training group only. **p < 0.01.

## 4 Discussion

In this study, we found that an 18-month meditation training was associated with decreased time spent in a state characterized by a weak connectivity between the DMN, SN, and ECN networks (“weakly connected” state), and increased time spent in a state characterized by strong connectivity between these three networks (“strongly connected” state). No such changes were found in the non-native language training and no intervention groups. However, there was no group x visit interaction, except for a trend for the “strongly connected” state in the subgroup of participants visiting the state (time > 0). We also found that the number of transitions between states increased only in the meditation group, with a significant group × visit interaction observed between the meditation and non-native language training groups.

Our findings thus indicate that the meditation training group switched significantly more between states post-intervention compared with baseline (pre-intervention), while no changes were observed in the non-native language training and no intervention groups. No previous study assessed the specific impact of meditation on this parameter. However, this result aligns with the literature, as switching between states has been interpreted as the signature of a conscious brain and is thought to be necessary for flexible cognition and behavior (Barttfeld et al., 2015;Deco et al., 2017;Tognoli & Kelso, 2014). Moreover, previous studies reported an association between a higher score of mindfulness and more transitions between states in adult and adolescent populations (Lim et al., 2018;Marusak et al., 2018). The authors argued that greater flexibility in transitioning between functional neural states may reflect greater capacity to switch the focus of attention back to the present moment from mind wandering. Building on this, we hypothesize that the increased number of transitions observed in the meditation group in our study reflects a more flexible brain capable of rapidly shifting between different states, including to disengage from mind wandering or maladaptive neural states. This fits with previous studies in expert meditators showing higher metastability (Escrichs et al., 2019) and increased long-range temporal correlation (Irrmischer et al., 2018), both indicative of an enhanced capacity for dynamic neural switching. Altogether, these findings support the idea that meditation training fosters greater neural flexibility, facilitating rapid shifts between tasks, mental states, or emotional contexts. This increased flexibility enables individuals to more effectively disengage from rigid or maladaptive neural states and develop a more adaptable and resilient neural system. In our previous studies assessing the links between dFNC metrics and risk and protective factors in aging, we found that fewer transitions positively correlated with lower brain perfusion in AD-sensitive regions, suggesting that a higher number of transitions would be associated with lower AD risk (Dautricourt et al., 2022). Additionally, we found that an increased number of transitions was significantly related to greater time spent in states associated with AD protective factors and less time in states associated with AD risk factors (Dautricourt et al., 2022). Altogether, our results suggest that an 18-month meditation training can both reduce a dFNC metric associated with AD risk factors and increase dFNC metrics associated with AD protective factors.

Additionally, our findings on the impact of the meditation training on the time spent in the different states (decrease for the “weakly connected” state and increase for the “strongly connected” state) tend to support a beneficial effect of meditation on aging. Indeed, firstly, in previous studies, the time spent in a similar “weakly connected” state has been found to be increased with age (Tian et al., 2018), as well as in various pathological conditions, including subjective cognitive decline (Chen et al., 2021), mild cognitive impairment (Du et al., 2020), and AD (Du et al., 2020). This state—characterized by lower functional connectivity across large-scale brain networks, reflecting a less synchronized brain activity pattern—could thus be a marker of brain vulnerability to aging and dementia. Consistent with this idea, in a previous study analyzing the baseline data of the same population, we found this “weakly connected” state to be associated with lower scores in early- and midlife cognitive activities and higher cardiovascular risks (low-density lipoprotein cholesterol) (Dautricourt et al., 2022), all known to be risk factors for AD. Our finding of decreased time spent in a weakly connected state with meditation training may indicate a reduction in fragmented brain network dynamics, linked to self-referential rumination, and reduced cognitive control (Brewer et al., 2011;Ganesan et al., 2024). This shift toward a more coherent organization may reflect a reduction in maladaptive, automatic thought patterns (Brewer et al., 2011). Notably, excessive rumination has been associated with overgeneral memory (Watkins et al., 2000), a cognitive pattern linked to depression and post-traumatic stress disorder, and considered a marker of maladaptive cognitive processing (Watkins & Teasdale, 2004). By diminishing time spent in this weakly connected state, meditation may foster a more adaptive cognitive processing mode, thereby potentially mitigating age-related cognitive decline.

Secondly, we also found the time spent in the “strongly connected” state to be associated with higher scores in early-life cognitive activities and global cognition, and reduced cardiovascular risks (body mass index or BMI). Thus, in opposition to the “weakly connected” state, this state was related to protective factors for AD. The “strongly connected” state is characterized by high connectivity between the DMN, SN, and ECN, indicating greater integration between networks. Our finding of increased time spent in a strongly connected state following meditation training may thus reflect enhanced interactions among the ECN, SN, and DMN. This increased connectivity likely facilitates improved conflict monitoring and cognitive control (via the ECN) over the DMN (Brewer et al., 2011;Hölzel et al., 2011), while also promoting better integration of internal stimuli processed by the SN with DMN activity. Such enhanced regulation could lead to an altered, more flexible and present-moment mode of self-processing, enabling a shift from narrative to experiential focus (Hölzel et al., 2011). This state is characterized by a body-centered, self-detached, and pre-reflective form of self-referential processing (Hölzel et al., 2011), potentially linked to cognitive defusion—a process that reduces rigid, self-focused thought patterns (Hayes et al., 2006;Hinojosa-Aguayo & González, 2022). In line with this interpretation, a recent study demonstrated that greater integration across networks is associated with cognitive defusion (Czajko et al., 2024)—a key mechanism of mindfulness (Segal et al., 2019;Zorn et al., 2021). Ultimately, this shift may help prevent age-related decline by reducing maladaptive cognitive patterns such as rumination (Watkins & Teasdale, 2001) and enhancing cognitive control, thereby promoting improved emotional regulation and overall well-being (Lutz et al., 2021). While we did not find a link between changes in brain states and cognitive defusion in our exploratory supplementary analyses, it is important to note that we did not expect that the changes in brain states occurring gradually over the 18 months would directly translate into changes in cognitive processes within this period. Future studies with longer follow-up periods are needed to clarify the links between dFNC changes and cognitive outcomes and their dynamics.

In contrast, the supplementary analyses revealed associations—in the meditation training group—between changes in brain state dynamics, specifically in the weakly connected state and the number of transitions, and both cognitive and psycho-affective measures. Specifically, a reduction in time spent in the “weakly connected” state was associated with lower cognitive decline and better episodic memory performance, suggesting that shifting away from this state may benefit cognitive preservation. Given prior evidence linking weakly integrated connectivity patterns to rumination and reduced cognitive control (Brewer et al., 2011;Ganesan et al., 2024), these results support the notion that meditation may help stabilize brain network dynamics in ways that promote attentional and memory processes. Additionally, a higher number of transitions between states was positively related to quality of life, social support, and meditation practice time. This aligns with the idea that a greater capacity for switching between brain states may reflect enhanced cognitive and emotional flexibility, supporting resilience, well-being, and social engagement. While these exploratory analyses provide preliminary insights, they remain secondary to our primary objective and should be interpreted with caution, as the results were not corrected for multiple comparisons.

Additionally, while the protective effects of meditation on Alzheimer’s disease and age-related decline are promising, these hypotheses remain indirect at this stage, and future studies with longer follow-up periods are needed to determine whether meditation directly influences dementia risk factors or primarily modulates brain states associated with higher or lower dementia risk.

Note that in the only study to date assessing the impact of an intervention including meditation on dFNC metrics, they rather found a decrease in a state characterized by strong connections between the DMN, SN, and executive network that resembles our “strongly connected” state (Mooneyham et al., 2017). Yet, this study is hardly comparable with ours as the intervention mixed meditation, physical, exercise, discussion, and readings and was tested in young adults. Moreover, their intervention lasted 6 weeks, and while the timing of response to meditation practice remains largely unknown, it has been suggested that meditation effects may vary with practice duration (Lutz et al., 2021). Given the novelty of this method, replication of our findings is still needed.

In light of these observations, we recognize that the mechanisms underlying distinct dFNC states are not yet fully understood. Future studies on acute dFNC changes during meditation inductions might help clarify these mechanisms by identifying short-term neural dynamics that contribute to the progressive changes observed over the course of extended meditation practice. Our results suggest that states characterized by strong or weak connectivity may offer more sensitivity than those primarily involving specific networks such as the DMN and SN. This emphasizes the need for deeper investigation to clarify whether the observed effects are due to differences in the stability or adaptability of these states, or whether global states are inherently more susceptible to interventions such as meditation due to their broader integration across multiple networks.

This research presents several strengths and limitations. A major strength of our study is the longitudinal design and the use of an RCT, which ensured rigorous testing of the meditation training’s effects and allowed for a valid assessment of its causal impact. The inclusion of both passive and active control groups further enabled us to evaluate whether the meditation training was superior to another cognitive activity. Additionally, the 18-month duration of the meditation training allowed us to observe effects that a shorter intervention might not reveal. Long-term interventions like this are rare, as most studies focus on shorter durations and, as such, represent a key strength of our study. While this extended period provided a unique opportunity to assess both immediate and sustained effects, it also presented challenges, including maintaining participant engagement, managing logistical considerations, and accounting for health-related events that may have impacted adherence or introduced variability. Moreover, the intervention’s reliance on participants’ motivation and adherence may have led to selection bias, with a sample predominantly composed of healthier individuals with higher education and cognitive reserve, potentially limiting the extent of intervention-related improvements. Despite these challenges, the longer duration enhances the ecological validity by better reflecting real-world conditions where individuals maintain meditation practices over time. However, the use of dFNC provided a novel methodological approach to assess temporal information about functional connectivity but it is still exploratory and requires replication in external cohorts to confirm the findings. Notably, most effects were small and there was no interaction between group and time on the time spent in states; future studies could test whether such differences might be detected in larger cohorts or pending further refinement of the method. Indeed, the sample size was not calculated for this outcome which could have prevented us from observing further results. Additionally, other factors not considered here such as the lifestyle of the participants or their genetic predisposition (e.g., Apolipoprotein E genotype) could have impacted the effects of the intervention on dFNC; further studies are needed to highlight the impact of these covariates. Also, future studies should assess the role of the specific active control task in the findings. While the effects appeared stronger between meditation and language learning compared with the passive control, we cannot exclude the possibility that the English intervention may have caused frustration among some participants, which could have contributed to disengagement. Although participants were generally well engaged in both interventions, these frustrations may partly explain the less favorable results observed in the English language training group. Furthermore, the effects observed within the meditation group do suggest a specific impact of meditation, but the differences observed in the post hoc comparisons between meditation and language training (but not between meditation and the passive control group) suggest that the control task may play a role in these results. Further studies are needed to investigate the specific impact of meditation and to better isolate the unique effects of meditation interventions on these biomarkers. Finally, our analyses focused on cognitive networks, but other networks could also be impacted by meditation, which might be explored in future studies.

In conclusion, dFNC offers novel insights into the temporal properties of functional brain networks and is particularly relevant to study the changes in brain network configuration associated with meditation training. In this study, we used longitudinal data to investigate changes in dFNC metrics associated with meditation practice. Our study demonstrated that an 18-month meditation training in older adults led to a significant increase in the number of transitions between brain states, an effect not observed in the non-native language training group and the no intervention group. The group × time interaction was significant for this measure, with a greater number of transitions in the meditation group than in the non-native language training group. Given that a higher number of transitions has been associated with lower risk for AD, this finding suggests a potential neuroprotective effect of meditation. Additionally, while we observed a decrease in time spent in a “weakly connected” state—linked to greater vulnerability to dementia—and an increase in time spent in a “strongly connected” state—linked to lower risk for dementia—these changes did not reach significance in group × time interactions. This suggests that, although within-group changes were evident, they did not exceed typical variability in these states. Altogether, our findings thus suggest that meditation training could both decrease and increase the time spent in states, respectively, associated with brain vulnerability and brain resistance to neurodegenerative diseases. However, these hypotheses remain preliminary, and the observed effects should be interpreted with caution. Further studies are needed to confirm these findings and explore the specific mechanisms underlying these effects. Future studies might identify the specific active mechanisms of meditation underlying these effects toward optimizing interventions.

## Ethics Approval and Consent to Participate

The Age-Well RCT was approved by the local ethics committee (CPP Nord-Ouest III, Caen; trial registration number: EudraCT: 2016-002441-36; IDRCB: 2016-A01767-44; ClinicalTrials.gov Identifier: NCT02977819). All participants gave their written informed consent prior to the examinations. The study was conducted in accordance with the Declaration of Helsinki.

## Supplementary Material

Supplementary Material

## Data Availability

Data are available on request following a formal data sharing agreement and approval by the consortium and executive committee. The data sharing request form can be downloaded athttps://silversantestudy.eu/2020/09/25/data-sharing/.
