# Supplementary material for: Effects of an 18-month meditation training on dynamic functional connectivity states in older adults: Secondary analyses from the Age-Well randomized controlled trial
Source: Imaging Neurosci (Camb). 2025 Jun 10;3:IMAG.a.33. doi: 10.1162/IMAG.a.33 (PMC12319754; doi:10.1162/IMAG.a.33)
Supplement: Supplementary Material [file imag.a.33_supp.pdf]

Supplementary Materials

Supplementary Methods

Interventions

Overview

The 18-month intervention period starts just after the randomization step for each of the three cohorts. During the study, participants are strongly encouraged not to practice the activity proposed in the other arms (groups). The number of teachers per class and their level of expertise are equal in both interventions. Participants are encouraged to participate in all those activities during the whole period of the intervention (i.e., 18 months).

For both the meditation and the foreign language training interventions, each weekly group session is divided into three parts: presentation of a theme, sharing, and formal practice. The first two sessions of each month include an equal share of these three parts (3x40min), session 3 includes more sharing (30/60/30min) and session 4 more practice (30/30/60). For both interventions, participants benefit from media (manual and audio) for their practice. The media (text, images, audio, video) and activities (alone, in pairs, in groups) rotation help to maintain interest and motivation. In addition, participants have to complete daily practice 20 runlogs at home and throughout the intervention on an electronic tablet to inform on their practice (duration, nature, difficulty and pleasantness level).

Monthly meetings are organized between the scientific investigators and the teachers of both interventions to optimize the intervention monitoring, and homogeneity. The teachers in charge of the intervention can also contact the scientific investigators at any time to keep them informed about any aspects related to the intervention or to the participants, to ensure that the care and follow-up of the participants are optimal.

Meditation intervention

The teaching content of the meditation intervention is shaped in 9 months dedicated to the teaching of mindfulness meditation followed by 9 months dedicated to the teaching of the

meditation on loving kindness and compassion. A new educational theme is introduced each month during the first session and is further developed, practiced and discussed in the other sessions of the month. In the mindfulness portion of the intervention, the participant learns to intentionally pay attention to his or her internal or external experiences in the present moment, without making any value judgment. The positive mental states (mental calm, compassion) or negative mental states (ruminations, difficult emotions) are observed without identifying or being absorbed by these experiences. The aim is that the present moment is lived in a more open and flexible way and is less dominated by mental conditioning that is a source of suffering. The mindfulness portion of this program is directly adapted from an 8-month mindfulness-based intervention especially designed for older adults and validated on a group of Francophone older adults.

The practice of kindness-based meditation (short for loving-kindness and compassion meditations) is aimed at improving the relationship with oneself and to the world by addressing in a more positive perspective emotions such as shame, self-criticism, or anger, and by developing gratitude and appreciation for positive experiences such as caring love or compassion. Building on the non-judgmental monitoring capacity developed in mindfulness meditation, the participants will learn to cultivate self- acceptance and kindness toward oneself for instance in relation to one's negative thoughts, distractions, difficult emotions, unpleasant physical sensations to foster appreciation toward positive qualities of one's mind (joy, contentment, …). The participants then learn to extend a similar attitude of care and loving-kindness toward their loved ones, toward neutral persons (e.g. stranger), or toward difficult persons, ultimately recognizing that the need for comfort, security, and happiness is shared by all living beings. There is one day of meditation with about 5 hours of practice during which participants immerse themselves more intensively into meditation practices.

English intervention

A positioning test is proposed during the inclusion visit at the end of the diagnostic battery (Table 1) to allow a precise assessment of the initial level of each participant. If the number of participants in the foreign language training group is higher than 15, subgroups of levels based on this test are established to facilitate teaching. The intervention consists of English exercises designed to reinforce each participant's abilities in understanding, writing and speaking. Sessions are held by mixing oral comprehension and expression activities to work in priority, the acquisition of new vocabulary and new grammatical structures. A large place is given to the recognition of the concepts discussed in previous courses. The progress of the participants is evaluated according to a training follow-up document and personalized or group-oriented help as needed. A day of practice on the Anglo-Norman island of Jersey is organized where participants have a mission to accomplish with information to obtain on different places, objects to find and items to buys.

Passive control group

Participants in the passive control group are requested not to change their habit and continue living as they used to before engaging in the study and until the end of V3. They are specifically asked not to engage in meditation or foreign language training.

Explained variance

Based on the elbow method, the ratio of within-cluster sum of square (WSS) to between-cluster sum of square (BSS) is 0.083 (Supplementary Figure S7). The explained variance can be calculated as follows: $\text{Explained Variance} = BSS / TSS$

where the total sum of square (TSS) is the sum of WSS and BSS: $TSS = BSS + WSS$

Thus, by substituting the values, the explained variance is derived from the proportion of the total variance that is explained by the between-cluster differences:

$$\text{Explained Variance} = BSS / TSS = BSS / 1.083 \times BSS \approx 0.923$$

Cluster sensitivity

To assess whether the number of states had an impact on the statistical analyses, we repeated our analyses using 3 and 5 clusters in the k-means clustering which was applied to the dynamic functional connectivity matrices to identify recurring functional connectivity patterns across time and subject space.

Non-parametric assessment of the intervention effect

We used permutation Anova to test for the group x time interaction and Wilcoxon test to investigate the within-group changes and the between-group differences. As Wilcoxon test do not allow regressing covariates, we used the rlm function from the MASS package to assess the association between the dFNC parameters and the covariates before using residuals to investigate the within-group changes and the between-group differences. We set the alternative hypothesis as "greater" or "lesser" following our hypotheses and indicated a diminution of the parameter from pre- to post-intervention or from group to group by adding a "*" before the reported effect size. All post-hoc analyses were corrected using bonferroni correction.

Links between changes in dFNC parameters and changes in risk/protective factors of dementia

We performed linear regressions corrected for age and sex between on one hand the delta between pre- and post-intervention of each dFNC parameter and on the other hand the delta for the same risk and protective factors as used in the study by Dautricourt *et al.* (2022) minus some like the Lifetime of Experience Questionnaire (LEQ) that were not available post-intervention (Dautricourt et al. 2022). As these analyses were exploratory, corrections for multiple comparisons were not applied.

Links between changes in dFNC parameters and changes in cognitive, psycho-affective, meditation composite scores, and practice duration measures

We performed linear regressions corrected for age and sex between on one hand the delta between pre- and post-intervention of each dFNC parameter and on the other hand the delta for a selection of cognitive, psycho-affective, and practice measures. For cognition, we used composite scores created by the Medit-Ageing consortium to represent broad cognitive domains that are impacted in aging: global cognition, executive functions, episodic memory and attention/processing speed. For psycho-affective measures, we included the following measures: depression, anxiety, emotion regulation, satisfaction with life, social support and quality of life. The meditation composite scores were based on a theoretical framework categorising meditation practices into attentional, constructive, and deconstructive types based on the cognitive mechanisms these practices primarily target or necessitate. These composite scores were the main secondary outcomes complementing the primary outcome measures of the Age-Well clinical trial, as published in Chetelat et al. (2022). Links were also assessed with the meditation practice, i.e. the total amount of practice time at home and the class attendance rate. As these analyses were exploratory, corrections for multiple comparisons were not applied.

Supplementary Results

States characteristics of sample subjects

S001: Weakly connected state = 62%, “SN-negatively connected” state = 13%, “Strongly connected” state = 21%, “DMN-negatively connected” state = 3%.

S002: Weakly connected state = 81%, "SN-negatively connected" state = 3%, "Strongly connected" state = 2%, "DMN-negatively connected" state = 14%.

S003: Weakly connected state = 43%, "SN-negatively connected" state = 16%, "Strongly connected" state = 16%, "DMN-negatively connected" state = 24%.

S004: Weakly connected state = 53%, "SN-negatively connected" state = 34%, "Strongly connected" state = 8%, "DMN-negatively connected" state = 5%.

Cluster sensitivity

Total time spent in each state: 3 states

The meditation group showed a decrease from baseline to post-intervention, in the total time spent in the "weakly connected" state (Cohen's d [95% Confidence Interval], -0.37 [-0.73, -0.02]), while no change were found for time spent in the "SN-negatively connected" state (0.07 [-0.34, 0.47]), and "DMN-negatively connected state" (0.32 [-0.07, 0.72]). Neither the non-native language training nor the no intervention group showed any change in the total time spent in any state (Supplementary Table S5). There was no significant group x visit interaction (Supplementary Table S6).

Number of transitions between states: 3 states

The meditation training group showed an increase, from baseline to post-intervention, in the number of transitions between states (Cohen's d [confidence interval], 0.33 [0.01, 0.75]). The non-native language training (-0.38 [-0.74, 0.03]) showed no change in the number of transitions between states while the no intervention group showed an increase (0.34 [0.03, 0.71]) (Supplementary Table S5). The group x visit interaction was significant ($p = 0.009$), and post-hoc analyses showed that the number of transitions increased more in the meditation than in the non-native language training group, as well as in the no intervention group compared to the non-native language training group (Supplementary Table S6).

Total time spent in each state: 5 states

When using 5 clusters, we identified the same four clusters as in our main analyses with the addition of a fifth cluster that closely resembles the “DMN-negatively connected” state.

Regarding our 4 clusters of interest, the meditation group showed an increase from baseline to post-intervention, in the total time spent in the "strongly connected" state (Cohen's d [95%

Confidence Interval], 0.47 [0.10, 0.94]), while no change were found for time spent in the “weakly connected” state (-0.2 [-0.57, 0.17]), the "SN-negatively connected" state (0.07 [-0.3, 0.44]), and "DMN-negatively connected state" (0.23 [-0.15, 0.62]). Neither the non-native language training nor the no intervention group showed any change in the total time spent in any state (Supplementary Table S7). There was no significant group x visit interaction (Supplementary Table S8).

Number of transitions between states: 5 states

The meditation training group showed an increase, from baseline to post-intervention, in the number of transitions between states (Cohen's d [confidence interval], 0.44 [0.02, 0.86]). Neither the non-native language training (-0.29 [-0.65, 0.07]) nor the no intervention group (0.11 [-0.27, 0.50]) showed any change in the number of transitions between states (Supplementary Table S7). The group x visit interaction was significant ($p = 0.019$), and post-hoc analyses showed that the number of transitions increased more in the meditation than in the non-native language training group, while no differences were observed between the meditation training and control groups or between the non-native language training and control groups (Supplementary Table S8).

Non-parametric analyses: Effect of the intervention

Total time spent in each state

Statistics of the non-parametric analyses are reported in Supplementary Table S9-S10. The meditation training group showed a decrease, from baseline to post-intervention, in the total time spent in the "weakly connected" state (effect size [95% Confidence Interval], *0.41 [0.14, 0.67]) and an increase in the total time spent in the "strongly connected" state (0.38 [0.08, 0.64]), while no change were found for time spent in the "SN-negatively connected" state (0.21 [0.01, 0.51]) and "DMN-negatively connected state" (0.08 [0.01, 0.39]). Neither the non-native language training nor the no intervention group showed any change in the total time spent in any state (Supplementary Table S9). There was no significant group x visit

interaction i.e. the change over time in the total time spent in the states was not significantly different between the groups (Supplementary Table S10).

Number of transitions between states

The meditation training group showed an increase, from baseline to post-intervention, in the number of transitions between states (effect size [confidence interval], 0.44 [0.16, 0.68]) while the non-native language training showed a decrease (*0.33 [0.04, 0.59]). The no intervention group (0.16 [0.01, 0.45]) showed no change in the number of transitions between states (Supplementary Table S9). The group x visit interaction was significant ($p = 0.007$), and post-hoc analyses showed that the number of transitions increased more in the meditation than in the non-native language training group (Supplementary Table S10).

Links between dFNC changes and risk/protective factors of dementia

The change in time spent in the “weakly connected” state was found to be negatively associated with the change of the PACC5 composite score ($p = 0.02$) such that reduced time spent in that state following the meditation intervention is associated with greater global cognition. The change in time spent in the “strongly connected” state was found to be negatively associated with the change of the BMI ($p = 0.02$) such that increased time spent in that state following the meditation intervention is associated with reduced BMI. No association were found with the other measures (Supplementary Figure S5).

Links between dFNC changes and cognitive, psycho-affective, meditation composite scores, and practice duration measures in the meditation training group

A reduction in the time spent in the “weakly connected” state was found to be associated with greater score in global cognition ($p = 0.02$) and episodic memory ($p = 0.02$) while an increase in the time spent in the “strongly connected” state was observed to be associated with poorer quality of life (environmental subscore: $p = 0.04$). An increase in the number of transitions following the meditation intervention has been found to be associated with increased quality of life (social: $p = 0.02$; psychological: $p = 0.01$) and greater social support ($p = 0.04$) as well

as greater meditation practice duration ($p = 0.03$; $p = 0.02$). No association were found with the other measures (Supplementary Figure S6 & table S11).

Supplementary Tables

Supplementary Table S1 – Anatomical description of the Stanford ICA template (Shirer et al., 2012)

ICA component	Regions	Networks
1	Precuneus<br>Posterior and middle cingulate cortices<br>Bilateral angular gyri	DMN
2	Precuneus<br>Posterior cingulate cortex<br>Retrosplenial cortex<br>Bilateral parahippocampal cortices<br>Bilateral angular gyri<br>Precuneus<br>Bilateral superior and middle frontal gyri<br>Bilateral middle occipital gyri	
3	Medial prefrontal cortex<br>Precuneus<br>Anterior, middle and posterior cingulate<br>cortices<br>Bilateral hippocampus<br>Bilateral angular gyri<br>Bilateral thalamus	
4	Bilateral anterior insula<br>Anterior cingulate cortex<br>Medial prefrontal cortex<br>Supplementary motor area<br>Middle frontal gyrus	SN
5	Bilateral posterior insula<br>Inferior and superior parietal gyri<br>Precuneus,<br>Middle cingulate cortex<br>Bilateral thalami<br>Bilateral putamen	
6	Right middle and superior frontal gyri<br>Right inferior and superior parietal gyri<br>Supramarginal gyrus<br>Right caudate	ECN
7	Left middle and superior frontal gyri<br>Left inferior and superior parietal gyri<br>Supramarginal gyrus	

DMN = default mode network, SN = salience network, ECN = executive control network

Supplementary Table S2: Characteristics of the four dFNC states

Characteristics	"Weaky connected" state	"SN-negatively connected" state	"Strongly connected" state	"DMN-negatively connected" state
Frequency	46%	21%	16%	17%
Frequency (pre-intervention)	48%	21%	15%	16%
Frequency (post-intervention)	43%	21%	17%	19%
Mean dwell time	22	12	9	9
Mean dwell time (pre-intervention)	23	12	8	8
Mean dwell time (post-intervention)	21	12	10	9
Transitions in	3.97	2.35	2.40	1.90
Transitions out	4.05	2.37	2.42	1.79
Transitions in (pre-intervention)	3.85	2.35	2.30	1.90
Transitions out (pre-intervention)	4.02	2.31	2.29	1.79
Transitions in (post-intervention)	4.10	2.34	2.51	1.91
Transitions out (post-intervention)	4.09	2.43	2.55	1.79
Number of participants spending time in this state (pre-intervention)	123	122	97	72
Number of participants spending time in this state (post-intervention)	124	89	103	77

This table summarizes the characteristics of the four dynamic functional network connectivity (dFNC) states identified in the study. For each state, the table presents the frequency (percentage of time spent in each state), mean dwell time (in number of windows), transitions in (average number of transitions into the state), transitions out (average number of transitions out of the state), and the number of participants who spent time in each state.

Supplementary Table S3: Statistics of the within-group changes of the linear mixed models in subgroups of subjects visiting each state (time > zero)

		Standardized estimated change					
Outcome	Visit	Meditation training	p	Non-native language training	p	No intervention	p
State 1	V3-V1	-0.44 (-0.88 to 0)	0.009	-0.02 (-0.45 to 0.4)	0.89	-0.19 (-0.63 to 0.24)	0.3
State 2	V3-V1	0.11 (-0.37 to 0.59)	0.51	0.04 (-0.41 to 0.49)	0.93	-0.12 (-0.35 to 0.59)	0.69
State 3	V3-V1	0.5 (0.01 to 0.99)	0.005	-0.18 (-0.67 to 0.31)	0.27	0.03 (-0.44 to 0.49)	0.77
State 4	V3-V1	0.23 (-0.37 to 0.83)	0.43	0.47 (-0.13 to 1.07)	0.28	-0.25 (-0.75 to 0.26)	0.27
Number of transition	V3-V1	0.52 (0.08 to 0.97)	0.007	-0.33 (-0.76 to 0.1)	0.09	0.18 (-0.25 to 0.62)	0.29

Values are expressed as Cohen's d (95% Confidence Interval). All analyses were adjusted for age, sex, and education. For within-group standardized estimated changes, positive values reflect improvement within a trial group from baseline (pre-intervention) to post-intervention; negative coefficients indicate the opposite. All p-value are adjusted for multiple comparisons with Tukey correction. Abbreviations: State 1 = "weakly connected" state, state 2 = "SN-negatively connected" state, state 3 = "strongly connected" state, state 4 = "DMN-negatively connected" state, V1 = baseline (pre-intervention) visit, V3 = post-intervention visit.

Supplementary Table S4: Statistics of the between-group changes of the linear mixed models in subgroups of subjects visiting each state (time > zero)

		Interaction Group x Visit			Difference in change : Meditation vs. Non-native language training		Difference in change: Meditation training vs. No intervention		Difference in change: Non-native language training vs. No intervention	
Outcome	Visit	F	P	PFDR	Mean (95% CI)	p	Mean (95% CI)	p	Mean (95% CI)	p
State 1	V3-V1	1.69	0.19	0.29	-0.43 (-0.87 to 0.01)	0.07	-0.23 (-0.68 to 0.21)	0.26	0.15 (-0.29 to 0.59)	0.5
State 2	V3-V1	0.09	0.91	0.91	0.22 (-0.29 to 0.73)	0.67	0.15 (-0.37 to 0.66)	0.84	-0.08 (-0.58 to 0.42)	0.81
State 3	V3-V1	4.19	0.018	0.07	0.84 (0.27 to 1.41)	0.006	0.56 (0.03 to 1.09)	0.06	-0.29 (-0.83 to 0.26)	0.3
State 4	V3-V1	1.57	0.22	0.29	0.14 (-0.64 to 0.92)	0.83	0.31 (-0.36 to 0.98)	0.19	0.18 (-0.49 to 0.84)	0.12
Number of transition	V3-V1	5.14	0.007	0.007	0.79 (0.33 to 1.24)	0.002	0.25 (-0.2 to 0.69)	0.23	-0.44 (-0.88 to 0.01)	0.05

Values are expressed as Cohen's d (95% Confidence Interval). All analyses were adjusted for age, sex, and education. For between-groups difference, positive values reflect a relatively greater improvement in a trial group (from baseline [pre-intervention] to post-intervention) compared with the specified reference trial group; negative coefficients indicate the opposite. Between-groups differences p-values are adjusted for multiple comparisons with Tukey correction. Abbreviations: State 1 = "weakly connected" state, state 2 = "SN-negatively connected" state, state 3 = "strongly connected" state, state 4 = "DMN-negatively connected" state, V1 = baseline (pre-intervention) visit, V3 = post-intervention visit.

Table S5: Statistics of the within-group changes in dFNC parameters from the linear mixed models (3 clusters)

		Standardized estimated change					
**Outcome**	**Visit**	**Meditation training**	**p**	**Non-native language training**	**p**	**No intervention**	**p**
State 1	V3-V1	-0.37 (-0.73 to -0.02)	**0.03**	-0.06 (-0.32 to 0.20)	0.73	-0.11 (-0.50 to 0.28)	0.56
State 2	V3-V1	0.06 (-0.34 to 0.47)	0.68	-0.02 (-0.31 to 0.26)	0.93	0.09 (-0.20 to 0.38)	0.59
State 3	V3-V1	0.32 (-0.07 to 0.72)	0.08	0.09 (-0.21 to 0.41)	0.69	0.03 (-0.30 to 0.35)	0.94
Number of transition	V3-V1	0.33 (0.1 to 0.75)	**0.05**	-0.38 (-0.74 to 0.03)	0.08	0.34 (0.03 to 0.71)	**0.05**

Values are expressed as Cohen's d (95% Confidence Interval). All analyses were adjusted for age, sex, and education. For within-group standardized estimated changes, positive values reflect improvement within a trial group from baseline (pre-intervention) to post-intervention; negative coefficients indicate the opposite. All p-values are adjusted for multiple comparisons with Tukey correction. Abbreviations: State 1 = "weakly connected" state, state2 = "SN-negatively connected" state, state 3 = "DMN-negatively connected" state, V1 = baseline (pre-intervention) visit, V3 = post-intervention visit.

Table S6: Statistics of the between-groups changes in dFNC parameters from the linear mixed models (3 clusters).

		Interaction Group x Visit			Difference in change : Meditation vs. Non-native language training		Difference in change: Meditation training vs. No intervention		Difference in change: Non-native language training vs. No intervention	
**Outcome**	**Visit**	**F**	**P**	**P_{FDR}**	**Mean (95% CI)**	**p**	**Mean (95% CI)**	**p**	**Mean (95% CI)**	**p**
State 1	V3-V1	1.09	0.34	0.66	-0.33 (-0.77 to 0.11)	0.17	-0.24 (-0.68 to 0.20)	0.24	0.04 (-0.39 to 0.48)	0.86
State 2	V3-V1	0.12	0.88	0.88	0.07 (-0.36 to 0.51)	0.71	-0.02 (-0.46 to 0.42)	0.93	-0.11 (-0.55 to 0.32)	0.65
State 3	V3-V1	0.82	0.44	0.66	0.23 (-0.21 to 0.66)	0.32	0.25 (-0.20 to 0.69)	0.23	0.05 (-0.38 to 0.49)	0.82
Number of transitions	V3-V1	4.94	**0.009**	**0.009**	0.58 (0.14 to 1.03)	**0.009**	-0.01 (-0.45 to 0.44)	0.96	-0.61 (-1.06. to -0.17)	**0.009**

Values are expressed as Cohen's d (95% Confidence Interval). All analyses were adjusted for age, sex, and education. For between-group differences, positive values reflect a relatively greater improvement in a trial group (from baseline [pre-intervention] to post-intervention) compared with the specified reference trial group; negative coefficients indicate the opposite. The p-values of the between-groups differences are adjusted for multiple comparisons with Tukey correction. Abbreviations: State 1 = "weakly connected" state, state 2 = "SN-negatively connected" state, state 3 = "strongly connected" state, V1 = baseline (pre-intervention) visit, V3 = post-intervention visit.

Supplementary Table S7: Statistics of the within-group changes in dFNC parameters from the linear mixed models (5 clusters)

		Standardized estimated change					
**Outcome**	**Visit**	**Meditation training**	**p**	**Non-native language training**	**p**	**No intervention**	**p**
State 1	V3-V1	-0.20 (-0.57 to 0.17)	0.32	-0.04 (-0.35 to 0.26)	0.90	-0.12 (-0.52 to 0.28)	0.62
State 2	V3-V1	0.07 (-0.30 to 0.44)	0.67	-0.03 (-0.32 to 0.26)	0.85	0.02 (-0.28 to 0.33)	0.90
State 3	V3-V1	0.47 (0.10 to 0.94)	**0.02**	-0.14 (-0.44 to 0.16)	0.45	0.19 (-0.14 to 0.52)	0.23
State 4	V3-V1	0.23 (-0.15 to 0.62)	0.23	0.18 (-0.17 to 0.53)	0.38	0.05 (-0.31 to 0.41)	0.82
State 5	V3-V1	-0.61 (-1.05 to -0.17)	0.003	0.04 (-0.33 to 0.41)	0.99	-0.14 (-0.53 to 0.27)	0.37
Number of transition	V3-V1	0.44 (0.02 to 0.86)	**0.019**	-0.29 (-0.65 to 0.07)	0.19	0.11 (-0.27 to 0.50)	0.49

Values are expressed as Cohen's d (95% Confidence Interval). All analyses were adjusted for age, sex, and education. For within-group standardized estimated changes, positive values reflect improvement within a trial group from baseline (pre-intervention) to post-intervention; negative coefficients indicate the opposite. All p-values are adjusted for multiple comparisons with Tukey correction. Abbreviations: State 1 = "weakly connected" state, state2 = "SN-negatively connected" state, state 3 = "strongly connected" state, state 4 = "DMN-negatively connected" state, V1 = baseline (pre-intervention) visit, V3 = post-intervention visit.

Supplementary Table S8: Statistics of the between-groups changes in dFNC parameters from the linear mixed models (5 clusters).

		Interaction Group x Visit			Difference in change : Meditation vs. Non-native language training		Difference in change: Meditation training vs. No intervention		Difference in change: Non-native language training vs. No intervention	
**Outcome**	**Visit**	**F**	**P**	**P_{FDR}**	**Mean (95% CI)**	**p**	**Mean (95% CI)**	**p**	**Mean (95% CI)**	**p**
State 1	V3-V1	0.21	0.81	0.91	-0.15 (-0.58 to 0.29)	0.52	-0.08 (-0.52 to 0.37)	0.72	0.06 (-0.37 to 0.50)	0.79
State 2	V3-V1	0.1	0.91	0.91	0.09 (-0.35 to 0.53)	0.66	0.05 (-0.39 to 0.49)	0.83	-0.05 (-0.49 to 0.38)	0.82
State 3	V3-V1	2.67	0.07	0.20	0.49 (0.04 to 0.93)	**0.02**	0.19 (-0.25 to 0.64)	0.38	-0.32 (-0.76 to 0.12)	0.16
State 4	V3-V1	0.26	0.77	0.91	0.06 (-0.38 to 0.50)	0.79	0.15 (-0.29 to 0.59)	0.48	0.1 (-0.34 to 0.54)	0.65
State 5	V3-V1	2.57	0.08	0.20	-0.48 (-0.92 to -0.03)	0.03	-0.36 (-0.81 to 0.08)	0.12	0.13 (-0.30 to 0.57)	0.52
Number of transitions	V3-V1	3.63	0.03	**0.03**	0.6 (0.15 to 1.04)	**0.008**	0.26 (-0.18 to 0.71)	0.22	-0.33 (-0.76 to -0.11)	0.15

Values are expressed as Cohen's d (95% Confidence Interval). All analyses were adjusted for age, sex, and education. For between-group differences, positive values reflect a relatively greater improvement in a trial group (from baseline [pre-intervention] to post-intervention) compared with the specified reference trial group; negative coefficients indicate the opposite. The p-values of the between-groups differences are adjusted for multiple comparisons with Tukey correction. Abbreviations: State 1 = "weakly connected" state, state 2 = "SN-negatively connected" state, state 3 = "strongly connected" state, state 4 = "DMN-negatively connected" state, V1 = baseline (pre-intervention) visit, V3 = post-intervention visit.

Supplementary Table S9: Statistics of the within-group changes in dFNC parameters from the non-parametric analyses

		Standardized estimated change					
**Outcome**	**Visit**	**Meditation training**	**p**	**Non-native language training**	**p**	**No intervention**	**p**
State 1	V3-V1	***0.41 (0.14 to 0.67)**	**0.003**	*0.09 (0.01 to 0.35)	0.38	*0.05 (0.01 to 0.41)	0.28
State 2	V3-V1	0.21 (0.01 to 0.51)	0.10	0.05 (0.01 to 0.36)	0.38	0.07 (0.01 to 0.38)	0.33
State 3	V3-V1	**0.38 (0.08 to 0.64)**	**0.009**	*0.20 (0.01 to 0.46)	0.09	0.04 (0.01 to 0.38)	0.41
State 4	V3-V1	0.08 (0.01 to 0.39)	0.31	0.04 (0.1 to 0.37)	0.39	*0.01 (0.1 to 0.38)	0.49
Number of transition	V3-V1	**0.44 (0.16, 0.68)**	**0.002**	***0.33 (0.04 to 0.59)**	**0.01**	0.16 (0.01 to 0.45)	0.15

*Values are expressed as effect size (95% Confidence Interval). All analyses were adjusted for age, sex, and education. For within-group standardized estimated changes, positive values reflect improvement within a trial group from baseline (pre-intervention) to post-intervention; negative coefficients indicate the opposite. All p-value are adjusted for multiple comparisons with Tukey correction. Abbreviations: State 1 = "weakly connected" state, state2 = "SN-negatively connected" state, state 3 = "strongly connected" state, state 4 = "DMN-negatively connected" state, V1 = baseline (pre-intervention) visit, V3 = post-intervention visit. * indicate diminution from pre- to post-intervention in the time spent in the state or in the number of transitions.*

Supplementary Table S10: Statistics of the between-groups changes of the non-parametric analyses.

				Difference in change : Meditation vs. Non-native language training		Difference in change: Meditation training vs. No intervention		Difference in change: Non-native language training vs. No intervention	
**Outcome**	**Visit**	**P**	**P_{FDR}**	**Mean (95% CI)**	**p**	**Mean (95% CI)**	**p**	**Mean (95% CI)**	**p**
State 1	V3-V1	0.29	0.58	*0.13 (0.01 to 0.33)	0.34	*0.08 (0.01 to 0.29)	0.74	0.01 (0.01 to 0.27)	1
State 2	V3-V1	0.90	0.90	0.14 (0.01 to 0.35)	0.32	0.11 (0.01 to 0.33)	1	0.02 (0.01 to 0.25)	0.50
State 3	V3-V1	0.06	0.24	**0.24 (0.04 to 0.44)**	**0.04**	0.14 (0.01 to 0.36)	0.30	*0.09 (0.01 to 0.33)	1
State 4	V3-V1	0.79	0.90	0.08 (0.01 to 0.29)	0.70	0.05 (0.01 to 0.28)	1	*0.03 (0.01 to 0.26)	1
Number of transitions	V3-V1	**0.007**	**0.007**	**0.35 (0.14 to 0.53)**	**0.001**	0.18 (0.02 to 0.39)	0.06	*0.12 (0.01 to 0.31)	0.15

*Values are expressed as effect size (95% Confidence Interval). All analyses were adjusted for age, sex, and education. For between-groups difference, positive values reflect a relatively greater improvement in a trial group (from baseline [pre-intervention] to post-intervention) compared with the specified reference trial group; negative coefficients indicate the opposite. Between-groups differences p-values are adjusted for multiple comparisons with Tukey correction. Abbreviations: State 1 = "weakly connected" state, state 2 = "SN-negatively connected" state, state 3 = "strongly connected" state, state 4 = "DMN-negatively connected" state, V1 = baseline (pre-intervention) visit, V3 = post-intervention visit. * indicate reduced time compared to the reference group.*

Supplementary table S11: Multiple linear regressions between change in each meditation composite score and each dFNC measures.

	Attentional		Constructive		Deconstructive	
	β	p	β	p	β	p
“weakly connected” state	-0.03	0.85	-0.13	0.44	-0.11	0.40
“Strongly connected” state	0.20	0.25	-0.02	0.90	0.06	0.36
Number of transitions	0.21	0.20	0.05	0.76	-0.01	0.49

Covariates: Age, Sex, Level of education

Supplementary Figures

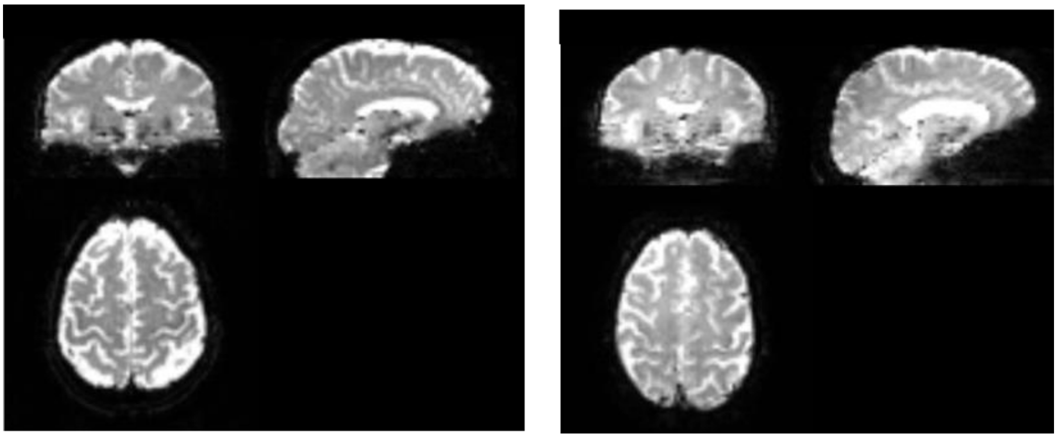

Figure S1: functional MRI images from two subjects of the Age-Well study before pretreatment.

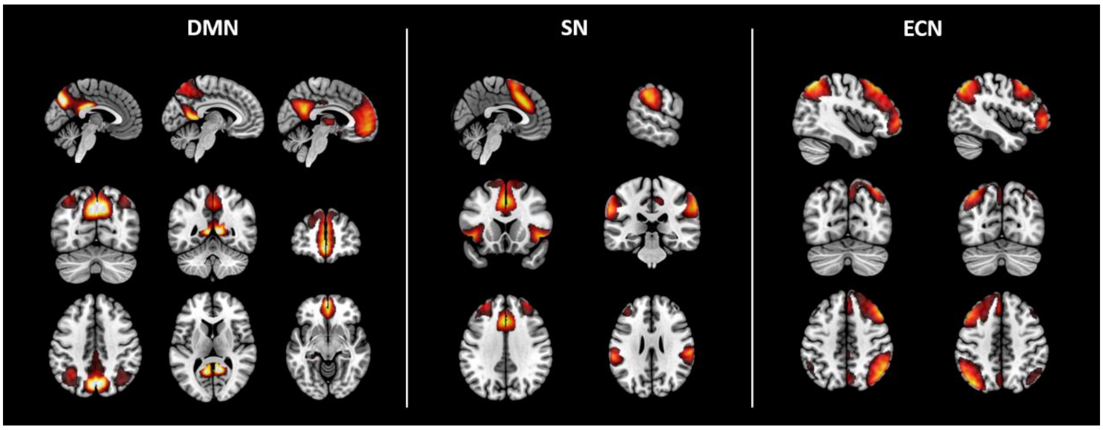

Supplementary Figure S2 - Intrinsic connectivity networks. *Representation of the independent component spatial maps obtained from the fully automated spatially constrained ICA with the Stanford atlas. The components are categorized according to their anatomical and functional properties in three distinct functional networks: the default mode network, salience network and executive control network DMN = default mode network; SN = salience network; ECN = executive control networks*

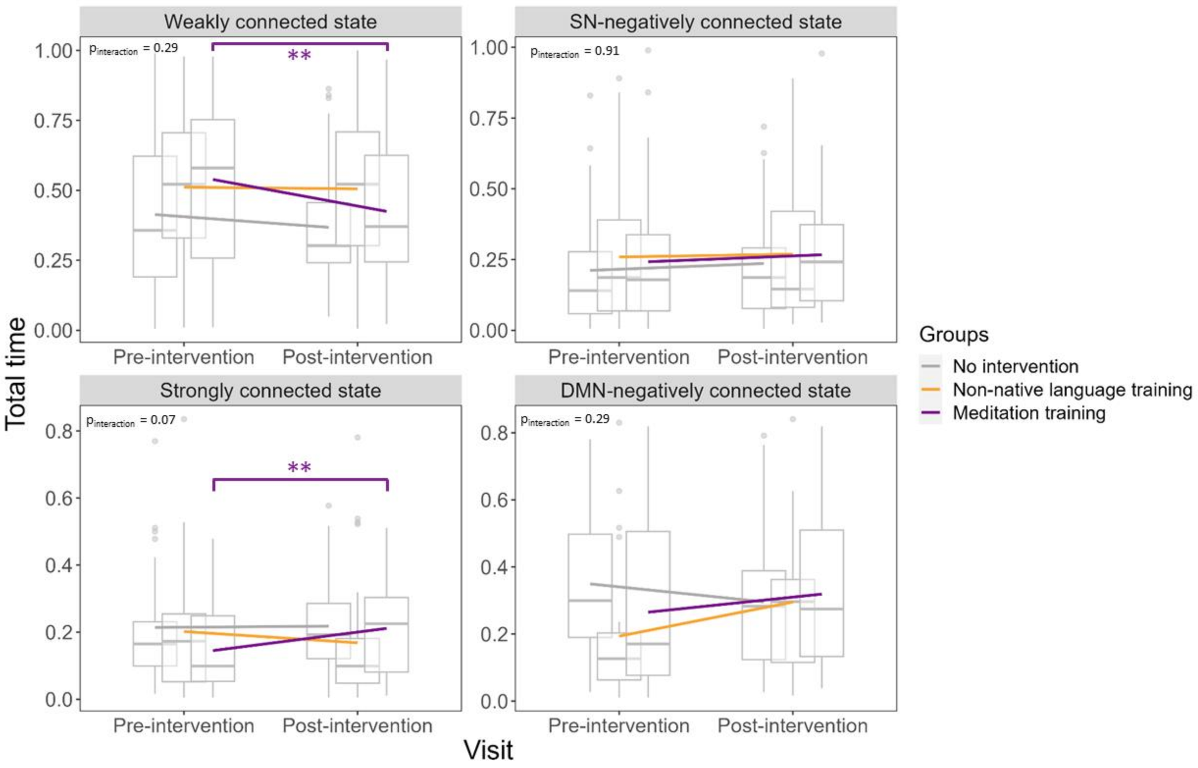

Supplementary Figure S3 – Total time spent in each state pre/post intervention in the subgroup of participants visiting the state (time>zero). *Linear mixed models show a significant interaction between group and visits for the total time spent in state 3, controlling for age, sex and education. The time spent in state 3 is significantly increased in post-intervention compared to pre-intervention in the meditation intervention group only. The time spent in state 1 is significantly decreased in post-intervention compared to pre-intervention in the meditation intervention group only. The total time in each state is expressed by number of repetition time (TR) (1 TR =2.4 seconds). ** p < 0.01.*

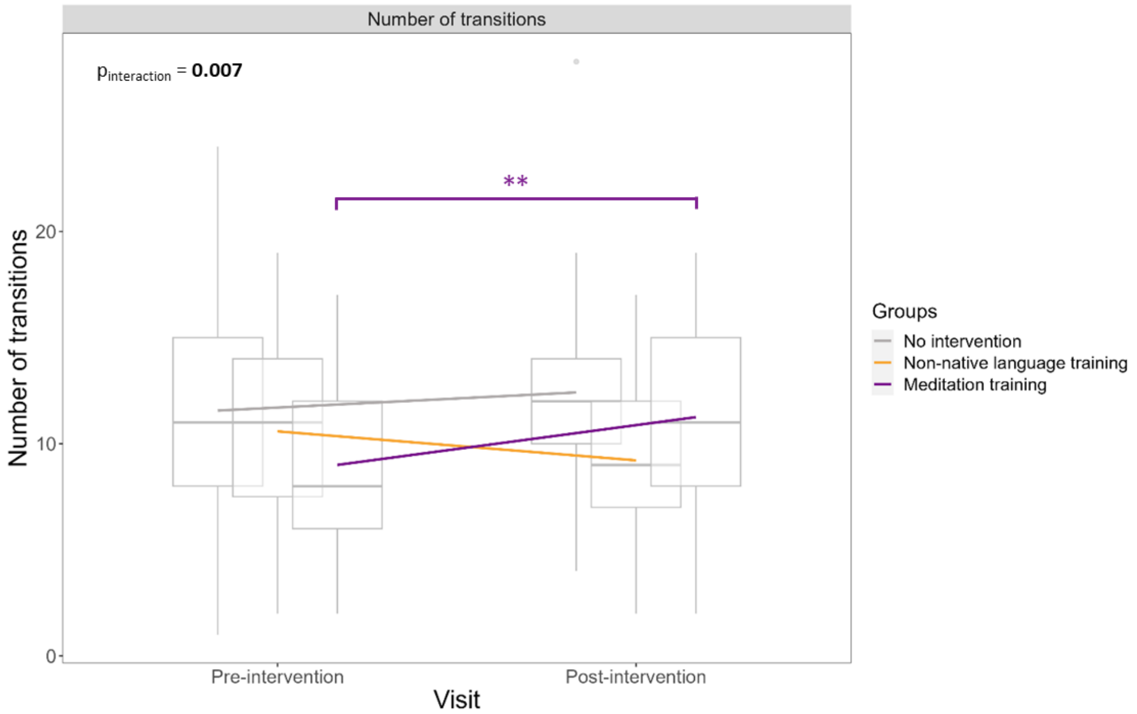

Supplementary Figure S4 – Number of transition between states pre/post intervention in the subgroup of participants visiting the state (time > zero). *Linear mixed models show a significant interaction between group and visits for the number of transitions, controlling for age, sex and education. The number of transition is significantly increased in post-intervention compared to pre-intervention in the meditation intervention group only. ** p < 0.01.*

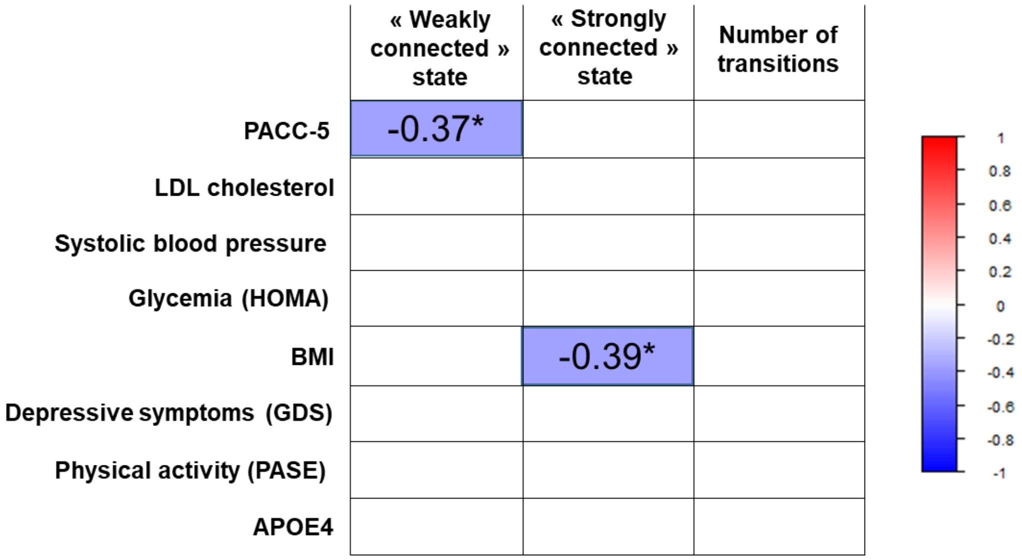

Figure S5: Matrix of correlation between changes in dFNC parameters and risk/protective factors of dementia in the meditation training group. *Positive correlations between variables are indicated in red and coefficient correlations are provided while negative correlations are indicated in blue. Blank spaces indicate non-significant relationships. * p < 0.05.*

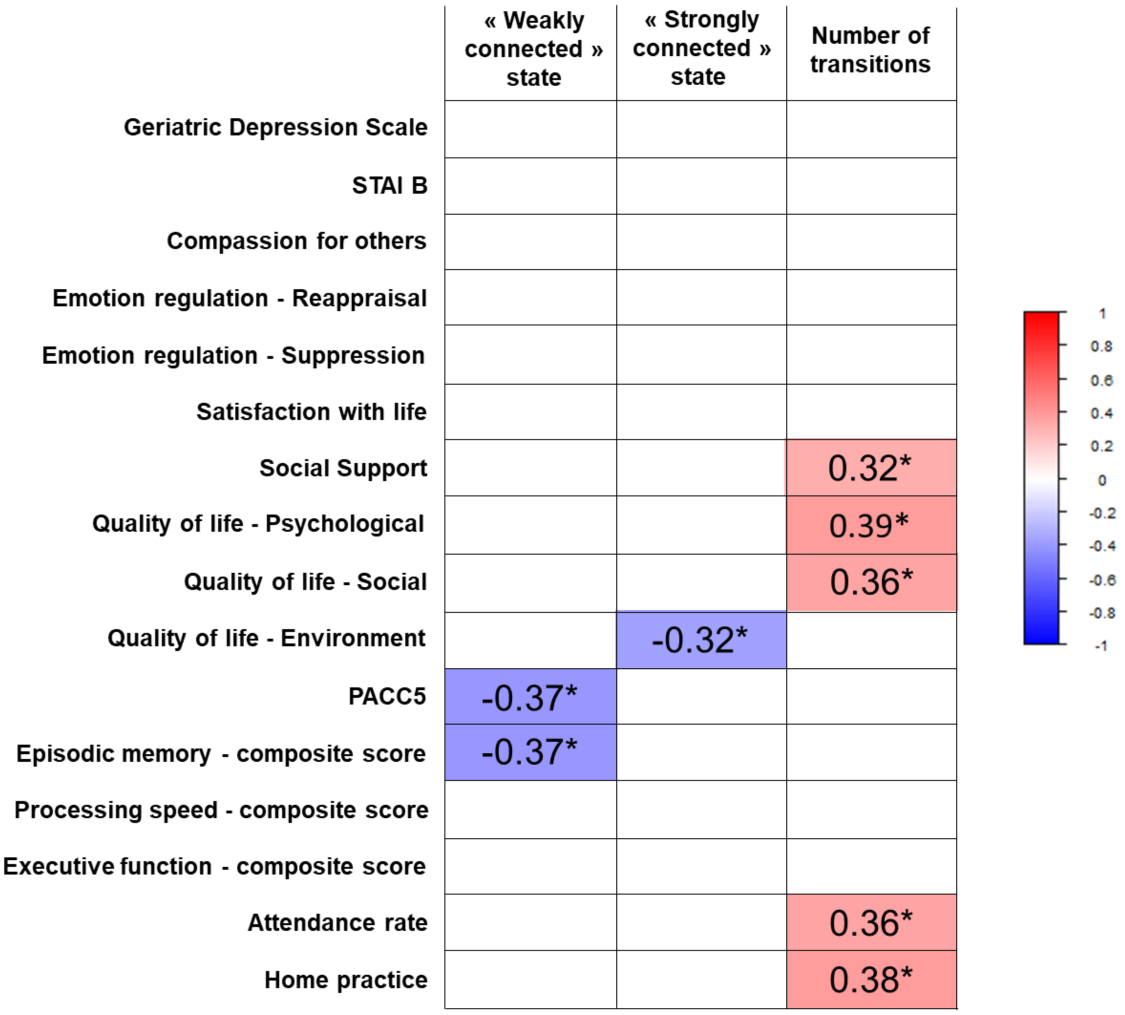

Figure S6: Matrix of correlation between changes in dFNC parameters and cognitive, psycho-affective, and practice measures in the meditation training group. *Positive correlations between variables are indicated in red and coefficient correlations are provided while negative correlations are indicated in blue. Blank spaces indicate non-significant relationships. * $p < 0.05$.*

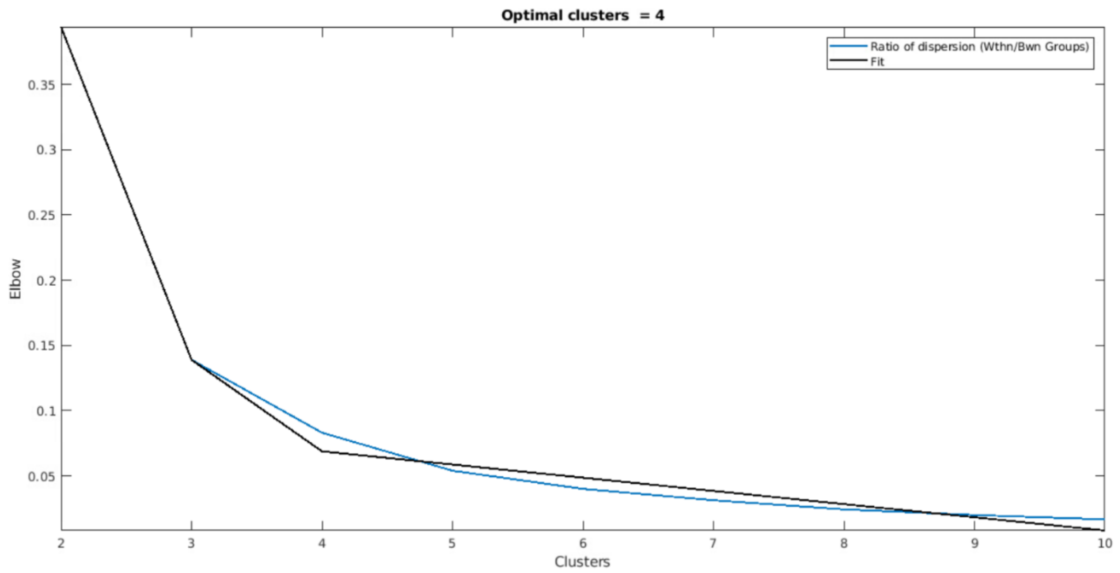

Supplementary figure S7: Elbow Curve of the WSS/BSS Ratio Across Varying Numbers of Clusters (k). *The figure depicts the ratio of the Within-Cluster Sum of Squares to the Between-Cluster Sum of Squares on the y-axis, plotted against the number of clusters (k) on the x-axis. The "elbow" point, where the ratio begins to stabilize, represents the optimal number of clusters for the analysis. Abbreviations: WSS = Within-cluster Sum of Squares, BSS = Between-cluster Sum of Squares.*
